# Fearing fear itself: Crowdsourced longitudinal data on Covid-19-related fear in Sweden

**DOI:** 10.1371/journal.pone.0253371

**Published:** 2021-07-01

**Authors:** Carol Tishelman, Jonas Hultin-Rosenberg, Anna Hadders, Lars E. Eriksson

**Affiliations:** 1 Division of Innovative Care Research, Department of Learning, Informatics, Management and Ethics, Karolinska Institutet, Stockholm, Sweden; 2 Center for Health Economics, Informatics and Health Care Research (CHIS) Stockholm Health Care Services (SLSO), Region Stockholm, Stockholm, Sweden; 3 Department of Government, Uppsala University, Uppsala, Sweden; 4 Uppsala Religion and Society Research Center, Uppsala, Sweden; 5 Regional Museum of Skåne, Kristianstad, Sweden; 6 School of Health Sciences, City, University of London, London, United Kingdom; 7 Medical Area Infectious Diseases, Karolinska University Hospital, Huddinge, Sweden; University of Haifa, ISRAEL

## Abstract

**Background:**

The Covid-19 pandemic has had unprecedented effects on individual lives and livelihoods as well as on social, health, economic and political systems and structures across the world. This article derives from a unique collaboration between researchers and museums using rapid response crowdsourcing to document contemporary life among the general public during the pandemic crisis in Sweden.

**Methods and findings:**

We use qualitative analysis to explore the narrative crowdsourced submissions of the same 88 individuals at two timepoints, during the 1^st^ and 2^nd^ pandemic waves, about what they most fear in relation to the Covid-19 pandemic, and how their descriptions changed over time. In this self-selected group, we found that aspects they most feared generally concerned responses to the pandemic on a societal level, rather than to the Covid-19 disease itself or other health-related issues. The most salient fears included a broad array of societal issues, including general societal collapse and fears about effects on social and political interactions among people with resulting impact on political order. Notably strong support for the Swedish pandemic response was expressed, despite both national and international criticism.

**Conclusions:**

This analysis fills a notable gap in research literature that lacks subjective and detailed investigation of experiences of the general public, despite recognition of the widespread effects of Covid-19 and its’ management strategies. Findings address controversy about the role of experts in formulating and communicating strategy, as well as implications of human responses to existential threats. Based on this analysis, we call for broader focus on societal issues related to this existential threat and the responses to it.

## Introduction/aim

The Covid-19 pandemic has taken an already tumultuous world by surprise, confronting society with new and dramatic challenges. The Covid-19 pandemic has already had unprecedented effects on individual lives and livelihoods as well as on social, health, economic and political systems and structures across the world [1]—effects to be dealt with now and for years to come. There is much to learn from people about their experiences living and working through the Covid-19 pandemic; experience-based data has the potential to inform how we deal with issues resulting from the disease itself and societal responses to it.

Museums have the potential to play a unique part in facilitating understanding of experiences of the pandemic, given their role in collecting, preserving, mediating, exhibiting and researching both material and immaterial cultural heritage, including testimonies about people and the human world of the past, present and future [2]. As cultural institutions, museums have been particularly affected by the limitations on social gatherings imposed in response to the pandemic internationally, but have in many cases adapted their activities to the new situation, e.g. by collecting Covid-19-related artifacts and experiences in a variety of forms (see e.g. [3–5]). The International Council of Museums advised museums to support community resilience during the pandemic through rapid response collecting and documenting the pandemic and its impact, a form of data collection called crowdsourcing here.

The term crowdsourcing was coined in 2006 by combining ‘crowd’ and ‘outsourcing’, and defined by Estellés-Arolas et al. [6] based on their 2012 review of the literature, as: *“a type of participative online activity in which an individual*, *an institution*, *a non-profit organization*, *or company proposes to a group of individuals of varying knowledge*, *heterogeneity*, *and number*, *via a flexible open call*, *the voluntary undertaking of a task”* (p. 197). *Crowdsourcing data* is considered a form of citizen science, in that citizens are involved in generation of new knowledge with benefits to themselves, science and society, among other characteristics [6, 7]. Crowdsourcing data has previously been proven useful in disaster responses [8], and the Covid-19 pandemic is not an exception in that it has generated a number of such initiatives, primarily focused on procuring clinical, epidemiological, and virological/genetic data (see [9]), although a lesser number of platforms, besides museums, have made efforts to collect narrative, experiential data [10].

The present study derives from a larger project, carried out in partnership with museums and public archives throughout Sweden, in which we use narrative rapid response crowdsourced data to learn from those in Sweden with a variety of different perspectives on Covid-19, with the goal of being better able to support individuals, families, health and social care staff, and communities in civic society. In this article, we explore how fear is expressed by the same individuals at two time-points, initially in Spring 2020 during the 1^st^ Covid-19 wave (T1), and again in autumn 2020 during the 2^nd^ wave (T2). We also examine if and how the same individuals’ descriptions of fear changed over time.

## Background

### The Swedish context

While the ambitions of the Swedish response to the pandemic are similar to those of most countries [11], there has been a good deal of international interest in the disease control strategies implemented in Sweden, as they differed from those of many countries in several ways [12, 13]. To contextualize the data presented here, we present background about the Covid-19 response in Sweden during the time the data underlying this study were generated, i.e. through November 2020.

The Swedish response has been described as less invasive than in many other countries, as it was based on voluntary, step-wise actions with no lockdown. Pre-and primary schools have generally remained open, as have stores, restaurants and most workplaces, although secondary schools and universities were on-line only in spring 2020. While there was some regulation in Sweden during this time, national strategy relied heavily on strong recommendations rather than legal, enforced restrictions [11]. Legislative regulation regarded international travel restriction, restriction of visits to residential care facilities for elders, size of public gatherings, as well as some requirements for restaurants, schools, etc [11].

Some specific recommendations varied over time, e.g. those related to protecting the population aged 70 and older, but most have been constant, e.g. repetition of the need to maintain good hand hygiene, keep physical distance to others, refrain from large gatherings, work from home when possible, avoid indoor social contacts, and limit non-essential travel, i.e. all emphasizing individual responsibility. The strategy has been based on eight key ambitions: mitigating burden by ‘flattening the curve’; protecting risk groups; safeguarding other health determinants and outcomes (e.g. by keeping schools open); ensuring availability of health/medical resources throughout the country; assuring that the rest of society remained functional; easing public concern through information on official websites and regular press conferences; explaining the reasons for measures taken; and implementing the ‘right measures at the right time’ [12].

The specificities of the Swedish response are in part due to the way health and social care are organized, differentiating responsibilities among three governance levels, with limited constitutional opportunity for minister intervention in the work of government agencies [11, 12]. The Swedish strategy rests on the principle of responsibility, in that the party normally responsible for a particular activity remains responsible for the activity in a crisis situation [14].

The most overarching level consists of the national government, which defines policy and governance for national agencies. While no single agency bears sole responsibility for dealing with Covid-19 related issues, several agencies have central roles. The Swedish Public Health Agency (PHA), has a mandate to provide information and recommendations on public health issues to key actors including the government, workplaces, associations and other agencies; this mandate includes responses to the pandemic [11]. Another key agency is the National Board of Health and Welfare (NBHW) with responsibility for assuring good health, social welfare, and quality health and social care in the country through guidelines and recommendations, and in this situation, assuring that there are sufficient beds for Covid-19 patients. The Swedish Civil Contingencies Agency (SCCA) is responsible before, during, and after crisis situations for issues concerning civil protection, public safety, emergency management and civil defense, that are not specifically delegated elsewhere [15].

The next level consists of 21 regions responsible for providing primary care and hospital-based outpatient and inpatient health care; each region also has their own unit for communicable disease control, with a high degree of autonomy and authority [12]. The third level is composed of the 290 municipalities responsible for provision of elder care, care for people with physical and mental disabilities, rehabilitation services, school health care, home care and social care [16]. Health and social care in this decentralized system are funded predominately through taxes, as well as support from national government, with comparatively low out-of-pocket costs for the individual [16, 17].

The PHA, the NBHW and the SCCA have been central in providing public information to date, jointly hosting press conferences held on all weekdays from March-August 2020, and twice weekly thereafter, with other instances invited to present information as deemed necessary. Elected officials played a lesser role in information provision in the first Covid-19 wave but have been more visible thereafter. However, while officials from the PHA argued that the Swedish Covid-19 strategy differed more in terms of rhetoric and language than in actual implementation [13], the strategy has been the subject of much critical debate in both Swedish and international media. Some of the most visible domestic critics formulated a group originally consisting of 22 researchers, who have been active in public debate, arguing for more stringent and forceful use of restrictive measures. However during the pandemic, Swedes were reported to have increased levels of what was already notably high trust in the government, societal institutions, and politicians, a trust that became less politicized during the pandemic’s first wave [18]. Sweden is also known for high levels of social trust, a phenomenon shared with other Scandinavian social welfare countries [19].

### Public responses to the pandemic: Existing literature

We searched the literature available through March 17^th^, 2021 for comparable research, reflecting perspectives on or responses to the pandemic from an adult general public. While there are a number of qualitative, interview-based studies of particular groups (e.g. [20]) and several surveys of the general public using pre-determined response alternatives [21–26], nearly all empirical research based on open responses from the general public was based on analysis of social media. Moss et al.’s [26] Norwegian analysis of 16 interviews with members of the public, recruited via their own networks and snowball sampling, is a notable exception; however these interviews were examined along with analysis of government communication about the pandemic to understand how people made sense of the meta-narrative.

Chakraborty et al. [27] and Imran et al. [28] used sentiment analysis to quantitatively explore Covid-19-related tweets, the latter from three pairs of neighboring countries, including Sweden and Norway. While the other country pairs showed high correlation between sentiments expressed, this was not the case in Scandinavia, where the tweets from Sweden evidenced positive sentiment for a longer time span than those from Norway. Flint et al. conducted two on-line surveys [29, 30], one of a high risk UK population and the other of a general population, predominately but not exclusively from the UK, about Covid-19 thoughts and behaviors, focusing on health-impact. Both cross-sectional surveys included several open, text-based questions, and even here, sentiment analysis was used to explore relationships to personality variables. Although sentiment analysis aims to understand human emotions expressed in text through natural language processing, it extracts and quantifies data, rather than qualitatively examining the content of the text-material itself.

A number of instruments attempt to measure Covid-19-related fear [21, 24], however those we found focus on the extent of fear, either in relation to signs of fear (e.g. clammy hands, sleep disturbances, heart palpitations [21]), general sense of unease [21, 24] or particular behaviors assumed to be indicative of fear (e.g. taking precautions against infection, following the news) [24]. Several indicators may however also be appropriate cautionary measures or responses, rather than maladaptive. Mertens et al. [24] did include a mandatory open question in their survey of 439 respondents recruited via social media, with nearly half from the Netherlands; while they illustrate the breadth in respondent’s major concerns, there is no further analysis of this qualitative data.

Gruchola & Slawek-Czochra [22] analyzed weekly reports of the quantitative “Eurobarometer: Public opinion monitoring in the time of Covid-19” survey from March-July 2020, with data from Sweden in two reports. While the Swedish sample did not report anxiety or experiences in response to questions about either health or social consequences or dangers, Swedes were among those from 24 countries reporting fear related to economic crisis, with a majority afraid of losing income, although these fears were not matched by reported experience. Unemployment was also the only finding presented in relation to fear, in a content analysis based on English and German language comments on three social media platforms in March 2020 [31].

Using different techniques, surveys have compared public perceptions in Sweden with Italy [25] and Norway [23]. In the former, both Italian and Swedish respondents were found to have a positive bias, reporting that they would be less likely than others to experience negative impacts of a range of other possible hazardous events including natural disasters, terror attacks, economic crises, domestic violence, and climate change, but only the Swedish sample demonstrated this optimism in relation to epidemics. The Swedish respondents also reported their knowledge and preparedness as less than that of responsible authorities, whereas the Italian sample reported feeling equal to authorities in this regard [25]. In an anonymous web-based survey distributed via Facebook in March-April 2020 in Norway and Sweden [23], respondents in both countries reported higher levels of worry about the national economy and about postponement of care for other conditions than Covid-19, than about their private economy. Responses in both countries were also strongly supportive of their own government’s manner of dealing with the pandemic, with the Swedish respondents expressing more trust in authorities than those in Norway.

## Methods

Co-author AH, curator at one regional museum in Southern Sweden, stimulated the collection of the unique crowdsourced data underlying this article by asking the general public to respond to three specific questions to document feelings in relation to the “galloping spread of the Corona-virus, Covid-19” [32]. These questions were: “What are you most afraid of right now?” (underlining not in original text), “What happens to you when you are afraid?”, and “Have you changed your way of life/how you act, because of fear of getting Covid-19?” The on-line form included information about gender, age and region of residence; provision of name and contact information was voluntary.

A press-release went out on March 18^th^, 2020 with a link to the on-line data collection, and information about this crowdsourcing initiative was broadly disseminated through social and mass media (newspaper articles, including translations to English and Arabic, radio interviews), particularly in the museum’s region, but with some national media attention as well. The call led to 364 responses by July 30^th^ with 278 submitted during March and 47 more in April 2020. One hundred ninety of the respondents included their email address for further contact.

The acuity of the Covid-19 situation in Sweden declined somewhat in the late summer of 2020, but a second wave became increasingly evident in the autumn and new local recommendations began to be put into effect in different regions from October 20^th^. Such recommendations were applied in the museum’s region—which had not been severely affected in the first pandemic wave—on October 27^th^, 2020. On October 26^th^, AH sent an email to all those who responded to the initial crowdsourcing data call and had provided an email address, asking them to email a response to the question, “What are you most afraid of right now?” (underlining not in original text), based on their perspective at that timepoint. Approximately 50 responses were received within a week of the request. A reminder email was sent out on November 18^th^. The database analyzed here consists of responses from 88 individuals at two time-points each.

### Ethical considerations

The data underlying the present study was collected and is being used in accordance with the International Council of Museums Code of Ethics [33]. In addition, the contributors received information and agreed to their responses being saved in the museum archive for use in future research, documentation and exhibitions, as well as information about the General Data Protection Regulation, and their right to access their data.

### Data analysis

Data analysis was inspired by Thorne’s interpretive descriptive approach [34], which provides an organizing logic, developed to generate understandings of complex phenomena with potential for use in practice from qualitative data. As it allows for a range of designs and methods to meet variations in context, situation and intent, rather than providing a set method, we present our analytic procedures in detail below.

Analysis was conducted by a team of two nurse researchers (a woman with dual background in social sciences (CT), and a man with dual background in natural science (LEE)) and a political scientist (JHR). CT and LEE initially read through the full data base from both time-points. LEE carried out preliminary coding, remaining close to the empirical data in the responses to both questions on what the individual was most afraid of at the first (T1) and second (T2) timepoints. CT and LEE discussed initial impressions from the naïve reading and initial coding, and together determined a preliminary coding scheme based on four levels, categorized as related to the individual, the individual’s closest circles, the health care system, and a broader societal level. It became clear that, in contrast to our expectations, submissions that described Covid-19 as a disease as the subject of most fear were relatively limited, and the bulk of the data instead related to what was initially conceptualized as a broad societal level.

At this point JHR joined the research team, given his background in political sociology and democratic theory. CT and JHR each (re)read the data in its entirety and individually determined preliminary themes in the data related to what was most feared on the societal level. The themes determined separately were discussed and found to be notably congruent. CT, JHR and LEE continued by discussing and determining key themes on the other levels, and together determined strategies for continued analysis. At this point, we recognized that few submissions about the health care system related to care provision per se, but instead appeared to represent fears related to societal structures; we therefore categorized such data in relation to the societal level instead. Preliminary findings were discussed with AH, the museum curator who originated and carried out data collection, and her feedback was incorporated into the final text.

Temporal aspects were initially summarized for each individual in terms of potential change of focus in the level of what was most feared, i.e. if fear was personal, in that it related to the respondent or those close to them, or on a broader, societal level. Thereafter we examined the content of the fear described for constancy or change. In examining change, we also referred back to the additional questions asked at T1 about how fear made the respondent feel and behave; as only one question was posed at T2, we attempted to assure that what we interpreted as change over time, was not seen previously in response to other questions at T1. We did find that several people described behaviors that might be interpreted as either indicating fear or reflecting appropriate caution (e.g. handwashing, avoiding close contacts with strangers). We changed categorizations only when responses were clearly related to what was most feared at either T1 or T2.

## Findings

Responses to questions varied, from just a few words to lengthier descriptions. Respondents ranged in age from 23–75, with median age 53. Seventy-four of the 88 respondents whose data were analyzed identified as women and the remainder men, with 61 residing in the same geographic region as the museum. In presenting the findings, we begin by describing patterns of change over time, followed by a section on the nature of fears at different levels; it should be remembered that these are responses to questions about what is MOST feared at the time. We generally refer to these levels as personal, i.e. relating to the individual and their closest circles, or as on a broader, societal level. Quotes are chosen to illustrate breadth and variation in responses. We have shortened some quotes, as indicated by (…), while maintaining their meaning, and have used lengthier, comprehensive quotes to illustrate several analytic points simultaneously.

### Change over time

In general, over half (n = 51) of these individual respondents were relatively consistent in terms of the level on which they focused their fears at both time points, although the specific content of their fear could change. Those who initially focused on personal or family issues generally continued to do so, whereas those who referred to societal issues at T1 also maintained this focus at T2, and those with composite reasoning integrating different levels at T1, generally continued to express their fears in this manner. A 57-year old woman (#350) exemplifies consistency in level of focus, in expressing fear about how she personally would be affected, but with some change in content. At T1 she writes *“…what can frighten me is that Covid-19 seems to be able to worsen so quickly*, *and then living alone and being bedridden at home alone*, *that thought can scare me”*. At T2, she remains concerned about her solitary life, although with less emphasis on Covid-19 as a disease, but rather on consequences of efforts to prevent its spread:

*(…) I want my spontaneous life back*, *where I could just decide that tonight I’ll go to the movies or this weekend I’ll take the bus out to the country*. *Even if we haven’t had a full-blown lockdown*, *it feels like one’s life has been put in isolation*, *that feeling isn’t good for how I feel*, *it’s meant a lot of time alone*.

Thirteen respondents appeared to change to more personal or family-related focus at T2, with many no longer including societal considerations apparent at T1 in their later descriptions. A woman in her mid-30s (#119) illustrates this, focusing on societal effects at T1, in a manner typical of many of who expressed a sense of pride in Swedish structures, but a degree of skepticism about her fellow citizens:

*I am mostly afraid that the already tense political situation in the world will escalate and it all will get worse*. *That people’s fear will lead to conflict instead of seeing it as we’re all in the same boat*. *We’re so lucky in this crisis*, *that we are able to be supported by the health care system and a government that still cares about people*. *In China they shot animals and welded doors shut*. *We have it better*, *but I’m afraid that it’s people’s fear that is going to be what topples us down in the ‘solitary is strong’* [refers to a Swedish expression “ensam är stark”, valuing independent individuals] *country of Sweden*.

However, at T2, her perspective had shifted dramatically. Her extensive response is written and spaced so as to be reminiscent of poetry, with strong emotional descriptions about what her fears mean to her. She also indirectly tells us that she works in the health care system, with an implicit criticism not present in her first response, expressed almost as an aside:

*Am I afraid?*

*Yes*, *is the short answer*. *The simple answer*. *The most concise*.

*But it’s also the response that lacks nuance most of all*.

*What am I afraid of?*

*I am afraid of becoming sick and losing time with my child*.

*I am afraid of dying from my child*.

*Because if I’m dead I’m not there when she needs me*.

*It frightens me not to be there when she learns to bicycle or swim*. *To not be able to see her write her own name for the first time or be able to hug her the first time she has heartache*.

*You asked me if I’m afraid*. *Yes*, *I sobbed as an answer without having health care staff testing* [referring to lack of access to Covid-19 tests for staf*f]*.

*I’ve had two sick colleagues and there I was*. *Stuffy nose*, *sore throat*.

*Who will tell her who I was if I’m not there?*

*Who will tell her that I danced while I cooked*, *that I lived on tangerines when I was pregnant with her and that every night I stroked her hair and whispered “[uses nickname]*. *My love*. *the one I love most”*

*Who will tell her that I climbed 26 meters down in a cave in Mexico*, *that I once talked about sex on the radio and that I most like to drink coffee at 6 in the morning next to a window cracked open?*

*Am I afraid?*

*Yes*.

*Am I infected?*

*No*.

Nineteen people broadened or shifted focus to a more societal level when describing their fears at T2, after initially describing fearing for themselves or their families. At T1, a middle-aged woman (#338) responded briefly, writing she was most frightened that she and her husband could die, leaving their children “*alone in this turbulent world”*. She both expanded upon and changed her expressions of fear at T2, at the same time saying she was less frightened. While her concerns are similar at both time-points and primarily described in relation to those close to her, at T2 she also relates this to the situation in society, referring particularly to the health care system, implications for the next generation as well as reflecting on the pervasiveness of existential uncertainty:

*What I’m most afraid of right now is naturally becoming so sick that I would need health care and wear down the already over-burdened health care system*. *That I would need care and maybe be on sick leave for a longer period (have colleagues who still aren’t themselves after having gotten infected in the spring) and be so sick that I don’t make it but become one of the statistics […]*. *I’m also afraid of infecting others in case I am infected*. *I’m even afraid of losing both my husband and children to this horrible infection*.

*Something else I’m afraid of is that my teenage sons won’t be able to have a memorable youth*, *like I had [*…*] They aren’t going to have that carefree time*, *no concerts*, *no parties*, *no traveling*. *Plus*, *that they’ll enter a reality where there aren’t jobs because of the economic crisis that is going to hit us*.

*But I’m not as panic-stricken as I was 7 months ago*, *even though every day entails a question of “if”*, *if one of us comes home infected*, *if we can celebrate Christmas with Grandma and Grandpa*, *if we can plan my […] birthday party next year*, *if our son can celebrate his graduation and if we are all still here until then*. *It’s become so much more obvious that life is so sadly fragile and short*.

Five respondents, of whom four were men, said they were not frightened at one or both time-points, sometimes commenting that the word itself was too strong; several others also commented that fear might be the wrong word, or that they weren’t afraid, but continued by describing strong concerns. A 65-year old man wrote he was not afraid of anything at T1 explaining: *“You can’t avoid a pandemic”*, maintaining that he was still not frightened at T2, *“just frustrated*. *You can’t influence anything (beyond being careful)”* (#337). One of three men who wrote they were not initially frightened first highlighted that fear was a choice, writing at T1: “*I’m not afraid of anything just now*. *I also don’t choose to be worried before something has happened for my own part*, *related to the virus”*. However, at T2 he said he felt concern, rather than fear, about other societal issues, referring to US politics and environmental issues (#115); both other men later wrote about fearing for family members’ health (#272, #336). A woman who was among the oldest respondents (#335) was unique in referring to knowledge she acquired as decreasing her fear between T1, when her primemost fear was that her older husband could become ill, noting that she was also more frightened of being infected by him than by anyone else, and T2, writing: *“I don’t feel especially frightened*. *I know much more about the pandemic now and know how to avoid being infected*. *I’m counting on there being limitations in social life for about another year or so and it feels ok…”*.

### The nature of fears expressed

As noted above, we categorized fears in relation to different levels. However, fear of duration could apply to both personal and societal levels, and vary in nature. This could sometimes relate to a specific object of fear lasting for an extended timepoint, or instead be expressed as overarching in relation to the pandemic, without further specification, as summarized by a woman in her 50s (#158) at T2: *“What I’m most afraid of now is that the pandemic will remain in the foreseeable future (…) I’m afraid that it’s going to be much worse before it gets better*. *If it gets better*. *Longing to see a light in the tunnel”*.

#### Individual and family levels = personal fears

A handful of people described being most fearful solely in relation to themselves, rather than also fearing for others as well; only one middle-aged woman (#123) reported fearing for becoming seriously ill and possibly dying at both T1 and T2, with no mention of others. Some people mentioned the effects of changed habits, often but not only, referring to social isolation. Mention of physical effects or work-related consequences for oneself were notably rare. This middle-aged woman, who defined herself as at high risk (#243), is one of few who described strong fear of physical consequences for herself and her son at T1; at T2 problems were described as even more severe, but less physical:

*What am I most afraid of?* *That I myself or someone close to me will get Covid-19 and die or have lasting negative effects*. *This is a daily worry that causes stress-related problems for me*, *and now with the pandemic I can’t get any psychiatric help despite referrals and EXTREME need*. *My body feels like it is about to give up and I stand here screaming for someone who can help me but am left all alone*. *It is horrible*. *I refuse to give up*, *but every day is a struggle with myself and my thoughts*.

A woman in her late 20’s (#306) is one of few who describe fearing direct side-effects of Covid-19, writing at T2: “*Most scared of having side effects or problems that come afterwards*. *Permanent loss of smell*, *decreased concentration*[…], *decreased lung capacity”*. However, this was only one aspect of her fears, as she continued by referring to societal issues, e.g. unemployment, isolation, and the lack of “*normal”* social behaviors. More often, fears about oneself were related to being a burden either to others or, as alluded to in several quotes above, on an already strained health care system. Fear of (inadvertently) transmitting the disease further was particularly common, as this middle-aged woman (#332, T1) concisely wrote *“…It is a disease that can be mild as a caress or as deadly as being hit by a club*. *You don’t want to get it*, *and you don’t want to give it*.*”*

Fears about the disease and its consequences were instead often expressed in relation to family members and older relatives and many, particularly at T2, described concerns about this in relation to the duration of the pandemic. This middle-aged woman (#5) who listed only societal concerns at T1, wrote at T2:

*I’m not afraid for myself*. *I’m concerned about not seeing people in my family who are spread out across the country*. *I’m afraid of not seeing my 90+ mother*. *I notice that she’s getting more and more depressed*, *isn’t eating so well*, *doesn’t care about the world around her anymore*.

While concerns about economic effects were generally expressed in broad, societal terms, some related economic issues more directly to family welfare, e.g. describing effects on family companies who had not yet received promised government support.

*The Societal level*. Data categorized as relating to the societal level are notable in the breadth of what was expressed as most feared, beyond the personal sphere. As a woman, one of the older responders (#18) succinctly explained:

“*I’m not so frightened for my own sake but feel worried that the whole society won’t be able to deal with all this stress*. *There is so much at stake for everyone*! *I’m especially worried about the health care system*.*”*

Fears on a societal level were generally more multi-faceted than those expressed on a personal level, and include fears about general societal collapse—with the health care system often a symbol of this as in the quote above, as well as changed social climate, changed political climate or even political order, and financial effects. Several people, in addition to or instead of addressing Covid-19 related fears, raised societal issues that might be considered as competing threats. The responses of a woman in her early 60s (#226) illustrate many of issues addressed in these data. At T1 she responds rather briefly, writing both about her hopes for responses from civil society, but also her fears about a changed political order, insinuating that there are other crises that deserve greater attention:

*The repercussions of what they call the Corona crisis*. *My hope is that people will realize how important it is that we help one another and are generous; considerate to each other*. *But it can also be the opposite*. *In times of difficult economic depression*, *dictatorships can arise*.

In her response at T2, this woman touches upon most of the issues raised in this database in relation to the societal level, although few other responses were as extensive or all-encompassing. She begins by addressing both national and global economic effects, and includes other crises confronting the world. Other societal threats are alluded to, as she mentions both climate change and political strife. She raises her fears about the global political order, before turning to how people will react to numerous challenges, practically and psychosocially, and how pandemic-related challenges will affect the social climate. She does however end on a positive note, with an expression of hope—albeit described as possibly naïve—and a smiley emoji.

*My answer to the question what I am most afraid of right now is that I’m worried about how the pandemic will hit society and world politics in general*. *All countries’ economies are affected*. *Are we going to have a new epoch of poverty in Sweden?* *Those generations alive today haven’t experienced the hardest times that Swedes went through during times of indentured servants when men and women (including children) toiled on farms and manors*, *in forges*, *mines and glassworks*. *On building sites…etc*.

*At the same time*, *we’re facing a global climate crisis*. *Hurricanes and flooding are increasingly common*.

*We have major powers like China and Russia and dictatorships like Belarus that threaten and oppress their populations*. *Will some major power*, *e*.*g*. *China*, *take the opportunity to take control of the world when countries are weakened as they deal with the pandemic?*

*With Corona spreading across the globe*, *people’s struggles for freedom and equality are hardly easier*.

*Are people going to be able to deal with these tensions?* *Will health care staff in hospitals*, *care homes*, *and home care be able to manage to keep on working?* *Some have lost their benefits after having gotten sick with a serious case of Covid 19*.

*How will people act when unemployment increases*, *when more people may have to leave their homes?* *When austerity belts have been tightened to the point of bursting and we might begin to suffer from lack of food*, *if household budgets don’t suffice*.

*Will people be able to cooperate so that we can help and assist each other instead of competing in a scenario like Russia after the fall of the Soviet Union*, *where up and coming oligarchs bought up companies that went bankrupt and became billionaires and wealthy at the expense of others*, *in a corrupt and criminal rule?*

*Will children and youth here in Sweden*, *manage to make up for missed schooling?* *(Everyone can’t succeed at distance-studies*)

*Will our way of life be more limited?* *More surveillance and control?* *Will isolation*, *loneliness and sorrow spread and do people in psychologically?*

*Will polarization and woes and horrors—and maybe even hostility—between people increase?* *Widespread fear in a population has never been good*.

*I can already see negative effects that I hadn’t counted on occurring when the pandemic was ‘in its infancy”*, *in March*. *People are starting to feel bad*.

*I harbor a hope that we people are smart enough to move towards each other instead of the opposite and realize that we need each other*. *Maybe naïve—but you have to believe in something ☺*

It should be noted that expressions of hope were not rare—both hope and hopelessness were expressed by many. As a man in his mid-50s (#151) summarized at the end of his T2 contribution: *“…The societal changes that followed/follow as a consequence are both frightening and hopeful*, *like most changes*:*)”*

While the contributions sent to the museum were generally supportive of the Swedish strategy for dealing with the pandemic, there are notable exceptions. A woman in her early 60’s (#38) is moderate in her reply at T1, writing: *“It’s uncertainty that’s worst*. *Is what we’re doing right or wrong?*[…]*”* One of the youngest responders (#334) is an outlier in her strong criticism of national policy at both timepoints. She is also one of the last responders at T1, writing in June, after several months of restrictions:

*That we sacrifice those who are old*, *who’ve payed tax their whole lives*. *We sacrifice them so that the pandemic won’t ruin things for future generations*. *That’s a horrible thought that terrifies me*. *It is also incredibly obnoxious and remarkable that many don’t follow the incredibly lax guidelines that the PHA have given us*. *The rest of the world is in quarantine and here they can’t even manage to keep distance and avoid social activities*. *That really makes me frightened*.

She further elaborates on her (relatively consistent) fears at T2, becoming more explicit about what she sees as a changing social climate and her skepticism toward policymakers.

*I am most afraid right now for an increased polarization in society in relation to how people react to the restrictions*. *It feels like there are those who mean you’re not allowed to do anything at all and then there are those who arrange parties*, *overnights at country cottages*, *etc*. *just like nothing has happened*.

*I think it’s frightening when I hear that health care staff refuse to use masks and say that masks don’t work*, *at the same time as other countries have orders to use masks in environments where it’s not possible to keep a distance*.

*I’m afraid of having to use public transport*, *to participate in group work at the university even though it doesn’t feel ok*. *Most of all*, *it doesn’t feel ok that the bus is full*, *and no one uses masks*, *because here in Sweden masks don’t work*, *which is strange*. *They work in every other country*, *why do Swedes do things so differently?* *Why doesn’t it work here?*

*I am afraid of the Swedish strategy which in my eyes is nonchalant and shows a lack of humility about what we don’t know*. *How many lives are we going to sacrifice?* *It’s scary*.

While this woman’s criticism of Swedish policy reflects a minority position in these data, her criticism of those who do not follow national policy is shared with many others contributing to this data base.

## Discussion

This explorative study is based on crowdsourced rapid response narrative data actively and voluntarily submitted to a Swedish museum at two timepoints during the 1^st^ and 2^nd^ waves of the Covid-19 pandemic. These submissions describe and often explain what was most feared in relation to the pandemic, from an individual perspective over time. We find that in this self-selected group of respondents, while fear is sometimes expressed in relation to oneself and one’s family, it is more often relayed in relation to a broad range of societal issues. The array of fears on a societal level included fear of general societal collapse which also encompassed the health care system, as well as a range of economic effects, and fears about how social and political interactions among people will be affected, with resulting impact on political order, including the suppression of other vital issues. Health-related fear, which seems to dominate much professional discourse, was less explicit and less common here than we had assumed would be the case. This analysis thus brings to mind the expression attributed first to Montaigne in the 16^th^ century [35] which became present-day axiom through Roosevelt’s first presidential inauguration speech in the U.S. in 1933 [36]. Roosevelt’s warning that “the only thing we have to fear is fear itself” is eerily relevant in relation to these data, given the focus of fears related to responses to, rather than the Covid-19 disease itself.

These findings thus differ notably from Mertens et al.’s results [24] from a social media-based survey in this respect, as they found the health of others was by far the most paramount concern (>46%), although we refrain from quantifying our results to avoid implying representativity of this self-selected sample. However, the substance of areas described as most feared and categorized here are well in line with those Mertens et al. [24] noted in response to their single open question.

Our analysis fills a gap in the research literature, which appears to lack subjective and detailed documentation of experiences from the general public, despite recognition of the widespread effects of Covid-19 and strategies for its management. Moss et al. [26] are a notable exception. While our findings bear similarities to those from Moss et al.’s [26], also selective, interview study, our data is unique in presenting data from the same individuals over time. However, many of the limitations in the nature of our data are also relevant in relation to Moss et al. [26]. Our data derive from people residing in Sweden, with a majority from one region, who have been active in contributing to a museum collection about the pandemic; Moss et al.’s [26] Norwegian interviews even reflected a limited political sphere, although participants varied in age and gender. Our contributors varied in age, but are predominately women, which may reflect those with most contact with cultural institutions and most engaged in issues related to health and health maintenance. This overrepresentation of women was also seen in much of the research we found despite varying data collection strategies [23, 24, 29, 30, 37]. Along with Moss et al. [26], Mondino et al.’s work [25] is an exception in their relative even gender distribution; in both cases this is based on researcher control in directly choosing interview subjects or using pre-existing panels for recruitment. One potentially positive aspect of our skewed gender balance is that, given the general underrepresentation of women in their collections, museums have a growing interest in gender issues [38] and documentation of engendered roles, particularly those of women, in contemporary life [39]. Our analysis provides insight into how Covid-19 affects daily lives of women (and men) in Sweden.

It should also be considered that while museum crowdsourcing theoretically allows all residents in Sweden to respond to issues of importance for them as individuals, regardless of background or ideology, it is clear that these data, which show relatively high levels of literacy and skill in expressing complex issues in writing, are biased toward those who appear well educated and integrated into mainstream Swedish society, and thus do not represent the diversity present in Sweden today. This limitation appears also relevant to Moss et al. [26] This lack of diversity should be considered, in part as demographic differences in Covid-19 disease spread and impact are well-acknowledged [40], but also, as Fitzpatrick et al found from the US, there may be an inverse relationship between the degree of fear reported and disease impact which may reflect structural inequalities in access to information [41].

Our findings indicate surprising homogeneity in ways we had not expected. While, as noted above, there are some expressions of uncertainty, there is notably strong support for Swedish policy and decisionmakers, despite the critical discourse showcased both in Swedish mass media and internationally [13]. This is in line with reports from the randomized sample in the Swedish SOM-Institute’s study, which compared their 2020 survey data with their pre-Covid-19 surveys, the last of which was immediately before the pandemic’s outbreak (Sept-Dec 2019) [18, 42]. They found heightened levels of trust in government agencies [18], including those responsible for Covid-19 information provision [42], morning newspapers and public service media [43], which appear relatively consistent across age, sex, education and political spectrum, with few exceptions, most notably related to less trust among those who identified with a right-wing populist party [18]. Our data do suggest some degree of increased skepticism over time, but do not allow robust conclusions to be drawn about this, although they provide insight into how people reason.

Epidemiologists and other experts have played an unprecedented public role in guiding, decision-making, and communicating official responses and strategies during the Covid-19 pandemic, not least in Sweden, and as noted above, particularly in the 1^st^ wave. The pandemic has thereby accentuated tensions in the role of experts and expertise in democracies, an ongoing debate since Plato’s time. On one hand, some warn that the pandemic has catalyzed the evolution of democracies into technocracies [44], whereas others suggest that the pandemic instead sheds light on the existing role of experts and expertise in present-day democracies [45], a role argued to have become increasingly significant over the past 50 years [46]. One scholarly concern about this ‘expertization’ is that the shift of policy decisions to the realm of science instead of being subject to political debate diminishes space for non-expert disagreement.

This theoretical discussion is interesting in relation to our data, as increased reliance on experts rather than politicians was not among the fears mentioned in these data, despite the wide array of fears related to political climate and order described as most salient. Such fears were often instead described in terms of increased polarization and xenophobia. While some contributors feared reduced freedom and expansion of political power, none expressed fear of a shift in power from elected officials to unelected experts, and indeed, as noted above, the relatively limited criticism of Swedish policy decisions in these contributions was in stark contrast to ongoing mass medial discourse. However, this somewhat surprising dearth of explicit criticism of the Swedish strategy among respondents should be considered in light of both a Swedish tradition of trust in officials [18, 19, 42, 43], but may also reflect that pandemic policy in Sweden was indeed largely framed and communicated by experts as grounded in scientific evidence rather than as a political issue. However, Eyal [47] warns in his discussion of “crises of expertise”, increased political reliance on expertise risks a parallel trend of increased skepticism toward scientific knowledge, i.e. a politicization of expertise. This can entail uncertainty in relation to expert knowledge, with determination of which experts are trustworthy becoming a political issue. A range of fears addressing polarization in these data may indeed reflect this tendency. The difference in trust along political lines cited above, may also reflect this phenomenon [18].

Despite polarization recurrently mentioned as a source of most fear, various types of polarization implicitly underlie many contributions in our data, which refer to e.g. generational conflicts in terms of a broadly-defined ‘we’ sacrificing the older population; ‘we’ in Sweden versus those outside Sweden who adhere to different policies; and perhaps the most salient, ‘we’ who follow recommendations and rules versus those who don’t. Polarization related to this latter issue may well be heightened as Swedish policy relied heavily on an individual responsibility, rather than enforced regulation. There appears to be a relatively homogenous, often implicit value system underlying most of the contributions. This is in line with the argument put forth in Terror Management Theory (TMT) that central mechanisms for keeping existential threats at bay are developing and maintaining common cultures, in the sense of worldviews of shared values, meaning and order which allow a sense of belonging [48], as well as enhancement of self-esteem [48]. TMT, based on Becker’s theories from the 1970s and tested in a wide range of situations since, postulates that one basic characteristic of humans is acting to avert threats to their existence and the terror such threats generate. As Solomon & Lawler [49] point out, in situations in which threats to mortality become salient, as arguably is the case during the Covid-19 pandemic [48], increased efforts are made to both consolidate and defend cultural beliefs, as well as bolster self-esteem. Both may lead to a pronounced sense of ‘we’ and ‘them’, which may well be one long-term effect of the Covid-19 pandemic worthy of consideration for the future.

In summary, the greatest fears expressed in this Swedish group of respondents are primarily related to policy responses resulting from professional and policy level fears about the dire health consequences of the pandemic. Based on this analysis, we join Moss et al.’s [26] call to policymakers to consider not only health-related aspects of the pandemic, but also how they address the emotional, behavioral and ideological continua of hope-fear, freedom-constraint and individuality-collectivity, respectively.

## Supporting information

S1 Data (XLSX)

S1 File (PDF)

## References

[1] World Economic Forum. COVID-19 Risks Outlook: A Preliminary Mapping and Its Implications. Geneva, Switzerland: World Economic Forum, 2020 20200519. Report No.

[2] Museilag (Swedish Museum Law), SFS 2017:563 (2017).

[3] Dickson A. How Will We Tell the Story of the Coronavirus? The New Yorker. 2020 20201209.

[4] Popescu A. How Will We Remember the Pandemic? Museums Are Already Deciding. NY Times. 2020 May 26, 2020.

[5] Science Museum Group. Collecting covid-19 2020 [20201217]. https://www.sciencemuseumgroup.org.uk/project/collecting-covid-19/.

[6] Estellés-Arolas E, González-Ladrón-de-Guevara F. Towards an integrated crowdsourcing definition. Journal of Information Science. 2012;38(2):189–200. doi: 10.1177/0165551512437638

[7] Haklay, Muki, Motion, Alice, Balázs, Bálint, et al. ECSA’s Characteristics of Citizen Science. 2020. 10.5281/zenodo.3758668.

[8] Riccardi MT. The power of crowdsourcing in disaster response operations. International Journal of Disaster Risk Reduction. 2016;20:123–8. 10.1016/j.ijdrr.2016.11.001.

[9] Leung GM, Leung K. Crowdsourcing data to mitigate epidemics. The Lancet Digital Health. 2020;2(4):e156–e7. doi: 10.1016/S2589-7500(20)30055-8 32296776 PMC7158995

[10] The Horizons Tracker. Crowdsourcing stories from the coronavirus pandemic 2020. http://adigaskell.org/2020/04/08/crowdsourcing-stories-from-the-coronavirus-pandemic/.

[11] Kavaliunas A, Ocaya P, Mumper J, Lindfeldt I, Kyhlstedt M. Swedish policy analysis for Covid-19. Health Policy and Technology. 2020;9(4):598–612. doi: 10.1016/j.hlpt.2020.08.009 32904437 PMC7455549

[12] Ludvigsson JF. The first eight months of Sweden’s COVID‐19 strategy and the key actions and actors that were involved. Acta Paediatrica. 2020;109(12):2459–71. doi: 10.1111/apa.15582 32951258 PMC7537539

[13] Irwin RE. Misinformation and de-contextualization: international media reporting on Sweden and COVID-19. Globalization and Health. 2020;16(1). doi: 10.1186/s12992-020-00588-x 32660503 PMC7356107

[14] Government Offices of Sweden. Strategy in response to the COVID-19 pandemic 2020 [20201211]. https://www.government.se/articles/2020/04/strategy-in-response-to-the-covid-19-pandemic/.

[15] The Swedish Civil Contingencies Agency (Myndigheten för Samhällsskydd och Beredskap MSB). 2020 [20210104]. https://www.msb.se/en/operations/ongoing-operations/coronavirus—covid-19/what-does-msb-do/.

[16] OECD. Sweden: Country Health Profile 20192019. 10.1787/2dcb7ca6-en.

[17] Lindqvist R, Alenius LS, Griffiths P, Runesdotter S, Tishelman C. Structural characteristics of hospitals and nurse-reported care quality, work environment, burnout and leaving intentions. J Nurs Manag. 2015;23(2):263–74. Epub 2013/09/21. doi: 10.1111/jonm.12123 . 24047463

[18] Andersson U, Oscarsson H. Institutionsförtroendet inte lika politiserat under pandemin. Göteborg: SOM-Institutet, 2020. https://www.gu.se/sites/default/files/2020-10/4.%20Institutionsf%C3%B6rtroendet%20inte%20lika%20politiserat%20under%20pandemin.pdf.

[19] Svendsen GLH, Svendsen GT. The Puzzle of the Scandinavian Welfare State and Social Trust. Issues in Social Science. 2015;3((2):90–9. 10.5296/iss.v3i2.8597.

[20] Bateman LB, Schoenberger Y-MM, Hansen B, Osborne TN, Okoro GC, Speights KM, et al. Confronting COVID-19 in under-resourced, African American neighborhoods: a qualitative study examining community member and stakeholders’ perceptions. Ethnicity & Health. 2021;26(1):49–67. doi: 10.1080/13557858.2021.1873250 33472411 PMC7875151

[21] Ahorsu DK, Lin C-Y, Imani V, Saffari M, Griffiths MD, Pakpour AH. The Fear of COVID-19 Scale: Development and Initial Validation. International Journal of Mental Health and Addiction. 2020. doi: 10.1007/s11469-020-00270-8 32226353 PMC7100496

[22] Gruchoła M, Sławek-Czochra M. “The culture of fear” of inhabitants of EU countries in their reaction to the COVID-19 pandemic–A study based on the reports of the Eurobarometer. Safety Science. 2021;135:105140. doi: 10.1016/j.ssci.2020.105140 PMC9759373 36570787

[23] Helsingen LM, Refsum E, Gjøstein DK, Løberg M, Bretthauer M, Kalager M, et al. The COVID-19 pandemic in Norway and Sweden–threats, trust, and impact on daily life: a comparative survey. BMC Public Health. 2020;20(1). doi: 10.1186/s12889-020-09615-3 33097011 PMC7582026

[24] Mertens G, Gerritsen L, Duijndam S, Salemink E, Engelhard IM. Fear of the coronavirus (COVID-19): Predictors in an online study conducted in March 2020. Journal of Anxiety Disorders. 2020;74:102258. doi: 10.1016/j.janxdis.2020.102258 32569905 PMC7286280

[25] Mondino E, Di Baldassarre G, Mård J, Ridolfi E, Rusca M. Public perceptions of multiple risks during the COVID-19 pandemic in Italy and Sweden. Scientific Data. 2020;7(1). doi: 10.1038/s41597-020-00778-7 33303742 PMC7729954

[26] Moss SM, Sandbakken EM. “Everybody Needs to Do Their Part, So We Can Get This Under Control.” Reactions to the Norwegian Government Meta‐Narratives on COVID‐19 Measures. Political Psychology. 2021. doi: 10.1111/pops.12727 33821063 PMC8014765

[27] Chakraborty K, Bhatia S, Bhattacharyya S, Platos J, Bag R, Hassanien AE. Sentiment Analysis of COVID-19 tweets by Deep Learning Classifiers—A study to show how popularity is affecting accuracy in social media. Applied Soft Computing. 2020;97:106754. doi: 10.1016/j.asoc.2020.106754 33013254 PMC7521435

[28] Imran AS, Daudpota SM, Kastrati Z, Batra R. Cross-Cultural Polarity and Emotion Detection Using Sentiment Analysis and Deep Learning on COVID-19 Related Tweets. IEEE Access. 2020;8:181074–90. doi: 10.1109/access.2020.3027350 PMC8545282 34812358

[29] Flint S, Piotrkowicz A, Watts K. Use of Artificial Intelligence to understand adults’ thoughts and behaviours relating to COVID-19. Perspectives in Public Health. 2021:175791392097933. doi: 10.1177/1757913920979332 33472547 PMC9047094

[30] Flint SW, Brown A, Tahrani AA, Piotrkowicz A, Joseph A-C. Cross-sectional analysis to explore the awareness, attitudes and actions of UK adults at high risk of severe illness from COVID-19. BMJ Open. 2020;10(12):e045309. doi: 10.1136/bmjopen-2020-045309 33376185 PMC7778740

[31] Olcer S, Yilmaz-Aslan Y, Brzoska P. Lay perspectives on social distancing and other official recommendations and regulations in the time of COVID-19: a qualitative study of social media posts. BMC Public Health. 2020;20(1):9. doi: 10.1186/s12889-019-8112-3 32560716 PMC7303937

[32] Regional Museum Skåne. Är du rädd? 2020 [cited 2020 20200625]. http://www.regionmuseet.se/ar-du-radd.html.

[33] International Council of Museums. ICOM Code of Ethics for Museums. Paris France: 2017.

[34] Thorne S. Intepretative Description. Second Edition ed. NYC: Routledge; 2016. 316 p.

[35] de Montaigne M. Complete Essays. Stanford: Stanford University Press; 1958.

[36] Roosevelt FD. 1st inaugural speech. http://historymatters.gmu.edu/d/5057/1933.

[37] Sandelin F. SOM-undersökningen om coronaviruset 2020- En metodöversikt. Göteborg: SOM-Institutet, 2020.

[38] Remer AE. Editorial. Museum International. 2020;72(1–2):1–7. doi: 10.1080/13500775.2020.1806586

[39] Oron E, Halevy M, Van Laar M, Giannakopoulou A. Action Plans Empower Museums to Address Gender Inequality. Museum International. 2020;72(1–2):68–79. doi: 10.1080/13500775.2020.1743058

[40] Burström B, Tao W. Social determinants of health and inequalities in COVID-19. European Journal of Public Health. 2020;30(4):617–8. doi: 10.1093/eurpub/ckaa095 32638998 PMC7454505

[41] Fitzpatrick KM, Harris C, Drawve G. The Consequences of COVID-19 Fear. Contexts. 2020;19(4):42–5. doi: 10.1177/1536504220977934

[42] Andersson U. Stort förtroende för Folkhälsomyndigheten och 1177 under coronapandemin. Göteborg: SOM-Institutet, 2020 Contract No.: 6. https://www.gu.se/sites/default/files/2020-10/6.%20Stort%20f%C3%B6rtroende%20f%C3%B6r%20Folkh%C3%A4lsomyndigheten%20och%201177%20under%20coronapandemin%20final.pdf.

[43] Andersson U. Medieförtroende i pandemitider. Göteborg: SOM-Institute, 2020.

[44] Horton R. Offline: The coming technocracy. The Lancet. 2020;396(10266):1869. doi: 10.1016/S0140-6736(20)32668-4 33308454

[45] Bylund PL, Packard MD. Separation of power and expertise: Evidence of the tyranny of experts in Sweden’s COVID‐19 responses. Southern Economic Journal. 2021. doi: 10.1002/soej.12493 33821054 PMC8014802

[46] Weingart P. Scientific expertise and political accountability: paradoxes of science in politics. Science and Public Policy. 1999;26(3):151–61. doi: 10.3152/147154399781782437

[47] Eyal G. The crisis of expertise.: John Wiley & Sons; 2019.

[48] Ahmed R, Ahmed A, Barkat W. Behavioral limitations of individuals for coping with COVID-19: A terror management perspective. Journal of Human Behavior in the Social Environment. 2020:1–22. doi: 10.1080/10911359.2020.1835778

[49] Solomon S, Lawlor K. Death Anxiety: The Challenge and the Promise of Whole Person Care. Springer New York; 2011. p. 97–107.

