# Supplementary material for: Fearing fear itself: Crowdsourced longitudinal data on Covid-19-related fear in Sweden
Source: PLoS One. 2021 Jul 1;16(7):e0253371. doi: 10.1371/journal.pone.0253371 (PMC8248701; doi:10.1371/journal.pone.0253371)
Supplement: S1 File — (PDF) [file pone.0253371.s002.pdf]

ID #	T1 submiss-ion date	1. Vad är du mest rädd för just nu? Förklara gärna (What are you most afraid of right now? Please explain)	2. Hur känns det/vad händer med dig när du blir rädd? (What happens to you when you are afraid?)	3. Har du förändrat din livsstil och hur du beter dig, p g a rädsla för att bli smittad? (Have you changed your way of life/how you act, because of fear of getting Ccovid-19?)	T2 fråga utskickad 20201026 Vad är du mest rädd för just nu? (What are you most afraid of right now?)	T2 Response after reminder sent 20201118	Gender	Birth year
5	20200318	Världsekonomin klimatet Att länder stängs ett hårdare klimat mot varandra	Osäker. Ibland uppgiven ibland aggressiv.	Jag kan inte hälsa på mina gamla föräldrar, jag handhälsar inte,	Jag är inte rädd för egen del. Jag är bekymrad över att inte få träffa personer i min familj som bor runt om i landet. Jag är rädd för att inte få träffa min mamma 90+ . Jag märker att hon blir allt mer deprimerad äter inte så bra bryr dig inte om omvärlden längre.		Kvinna	1956
18	20200318	Jag är inte så rädd för min egen del men känner obehag för att hela samhället inte ska klara av dessa påfrestningar. Det är så mycket som står på spel för alla människor! Särskilt vården är jag orolig för.	Det känns som hela samhället stannar av och jag som är 70 plus (frisk för övrigt) har särskilda restriktioner. Jag har lite svårt att ta detta till mej som gör att det känns overkligt/obehagligt.	Har slutat med alla mina aktiviteter (är ganska aktiv med både gym, teaterbesök, sociala aktiviteter m.m. Handla gör jag fortfarande en gång i veckan. Tvätta händer m.m har ökat avsevärt.	Jag är mest rädd fört att smittan ska pridas ännu mer och att vi blir mer isolerade från familj o vänner.<br>Det blir som att leva i en bubbla när inte de vardagliga/naturliga kontakterna med andra människor upphör.		Kvinna	1947

20	20200318	For at samfunnet kollapser pga konkurser og panikk.	Ingenting	Ikke pga redsel for å bli smittet, for det tror jeg vi må igjennom og for de fleste er det kun en vanlig influensa. Men følger myndighetenes råd for å støtte opp om sårbare grupper og helsepersonell og annet kritisk personell som må jobbe.		Det jeg er mest redd for nå er ikke corona men konsekvensene av tiltakene.<br>Hvor mange selvmord, tap av jobb, hus og skilsmisser.<br>Jeg tror det er større sjanse å dø eller få varige skader av å gå ut på gaten eller å kjøre bil, og det gjør alle hver dag, enn at det skjer av corona.<br>Mer redd for massehysteri frambragt av myndigheter og medier.<br>Beskytt de gamle og syke med å isolere dem fra andre og la andre leve normalt og heller ta sjanse å bli syk.<br>Når corona er over dukker det nok opp noe nytt. Kanskje det er jordens måte og ordne opp i våres største problem, overbefolkning 😉	Kvinna	1960

24	2020031 8	Jag är mest rädd för att andra människor och föräldrar i skolan , nu och i flera veckor INTE är och har varit rädda alls och därmed oaktsamma och egoistiska. Lite snor hit. Lite halsont dit - äh - denna hysteri-attityden.	Jag är och har varit arg. Jättearg sedan slutet av februari när jag kände det som att jag var den enda i Sverige som förstod vad som skulle hända. Jag är fortfarande arg - men äntligen har folk börjat anpassa sig lite och lite enkla riktlinjer är på plats. Det tog bara 2 månader.	Jag bunkrade mat och köpte handsprit, munskydd och hämtade ut mina mediciner redan i slutet av januari. Jag visste vad som skulle ske. Jag är bara förvånad över hur naivt dumma resten av befolkningen är.		Just nu är jag mest rädd för att all ilska, stress,oro och ångest som jag levt med under merparten av pandemin har fått en negativ påverkan på min kropp och utlöst nya kroniska men. Pandemin är jag inte lika orolig för längre eftersom jag behandlar min oro med antidepressiva mediciner sedan sommaren.”	Kvinna	1979
25	2020031 8	Ekonomisk krasch med depression och massarbetslöshet som på 30-talet. Jag är rädd även för vad det skulle kunna få för motsättningar mellan grupper. Sedan finns ju så klart den personliga rädslan för att någon av mina älskade ska drabbas av svår sjukdommen och kanske inte få rätt hjälp i en överbelastad hörapparat.	Jag känner näatan hela tiden en malande oro som lägger sordin på tillvaron. Osäkerheten inför framtiden avspeglar sig i varje beslut - ska vi vänta med de renoveringar och investeringar vi planerat - tänk om vi blir arbetslösa?	Inte så mycket än så länge. Det handlar mest om att följa de direktiv som ges och respektera restriktionerna. Pojkarnas fotbollsträning är inställt och förra veckan höll vi vår sjuåring hemma från skolan för att han hostade och nös några gånger.		En av sakerna jag är rädd för just nu är att någon av mina närstående ska bli allvarligt sjuk och kanske rentav dö, eller få men på lång sikt av orsaker som vi idag inte känner till. Mer tänkbart och lika skrämmande är tanken på en överbelastad sjukvård som inte fungerar pga Covid-19 - att människor inte får den vård de behöver, oavsett orsaken till deras behov.	Kvinna	1979

27	20200318	Jag är mest rädd för undergångskänslan hela samhället tycks ha drabbats av. Harmageddon. Jag är 70 år. Blev det 2 mars i år. Jag drabbades av "Asiaten" 1957. Min mamma fick "Hongkong" 1968. Av Asiaten minns jag inte mycket, men mamma fick Hongkong på julen då vi skulle fira jul hos min systers familj i Danmark. Pappa och jag fick åka själva. Sen kom Fågelinfluensan och Svininfluensan och jag har nog missat ett par emellan, men ingen har skapat en sådan undergångsstämning som Covid-19. Jag är rädd att vår svärsons företag går omkull. Ett familjeföretag med 6 anställda där han just fått säga upp 3 av dem. Vår dotter ringde och grät av sorg. Av miljarderna som regeringen mfl utlovat har han inte sett röken.			Jag är fortfarande rädd för att bli smittad och/eller att min man ska bli sjuk. Efter vårens och sommarens skandaler, där äldre i eget boende blivit nekade sjukhus-/intensivvård vid Covid-19, enligt en uppmaning ifrån Socialstyrelsen tidigt i våras och att äldre på äldreboenden, med hemtjänst och personer med LSS, också blivit nekade ens ett besök av läkare, utan via telefon ordineras "palliativ vård ", är jag ännu räddare att få Covid-19. Covid-19 yttrar sig bl a som andningssvårigheter. Att då ordineras morfin och Midazolam enligt Socialstyrelsen, som båda är andningshämmande, är inget annat än aktiv dödshjälp. "Eller något ännu värre" som geriatrikern Yngve Gustavsson sagt i media. Patienterna har inte ens fått syrgas, som kan faktiskt rädda livet, utan de har kvävts och dött en död, som i många fall skulle ha kunnat förhindrats med syrgas, antibiotika, dropp och antiinflammatoriska läkemedel<br><br>Detta är skälen till att 70%av de som dött i Covid-19 i Sverige har varit 70+ och har			Kvinna	1950

					bott i särskilt boende, haft hemtjänst eller omfattats av LSS.<br>Det har pågått ett upprop på Facebook, där en vårdcentralsläkare, Jon Tallinger, som uppmanats av Socialstyrelsen att "vårdplanera " sina äldre patienter för att förmå dem att avsäga sig sjukhusvård, livsuppehållande åtgärder vid Covid-19 mm, har tvärvägrat att göra detta och istället skrivit och protesterat överallt där han kunnat göra sin röst hörd. Bl a en anmälan om "Genicide"- äldremord till Europadomstolen med en lång rad vittnesmål från anhöriga och vårdpersonal i skrift och videolänkar bifogade.<br>Han anmäldes till IVO. Sades upp från sitt jobb och har nu emigrerat till Danmark. Allt det som Socialstyrelen sänt ut till Regionerna, vårdcentraler mfl skulle tystas ner. Men kvar finns de utförliga, daterade uppmaningar från dem i skrift, som snart blivit virala och som uppmanade regioner och vård att behandla äldre som beskrivet ovan. De skulle få " den bästa vården" I hemmen, innebärande en kvävningsdöd med morfin			

					och benzoediazepiner. Jag har läst dessa brev ifrån Socialstyrelsen och från bl a Region Stockholm och Region Gävleborg och chockats över hur vårdpersonal, tydligen med gott samvete, har kunnat följa denna beskrivna utsortering av människor, bara för att det inte skulle bli för stor belastning på IVA. Det är fasansfullt. Och denna "sorteringslista" finns ännu kvar och ska följas.<br><br>Äntligen har svensk media och IVO börjat utreda anmälningarna som strömmat in till dem ifrån anhöriga och sjukvårdspersonal om äldremorden. Det har lett till att politikerna har börjat reagera och ställa krav på bättre äldreomsorg. Något vi inte haft sedan decennier. Jag vet, eftersom min mamma, sjuksköterska som jobbade där, och som gick i pension 1978, kämpade i motvind mot idiotiska politikerbeslut, bl a de s k "Ädelreformen" där alla äldre skulle bo I eget boende och få omsorg där. Vilket resulterade i att allt som liknade sjukvård, bl a syrgas, togs bort. För "de bodde ju hemma"...			

					Till min rädsla har även kommit ilska. Vi som är 70+ har under hela pandemin fått isolera oss, börja köpa dyrare mat på nätet, ta dyr taxi ensamma, istf att ta buss eller tåg, undvika barn, barnbarn, kyrka, bio, teater, fester mm, bara för att yngre skall kunna leva ungefär som vanligt. Vi har fått bära hela ansvaret för att inte sprida smitta. FHM har skrämt upp oss och gjort oss till ensamma, eremiter. Och nu, när vi står inför en andra våg, så släpper FHM på reglerna för även oss! Och yngre tar fortfarande inte minsta ansvar! En ung tjej blev intervjuad i TV. Tyckte att hon ju "inte kunde stänga in sig hemma. Vad ska man göra där, liksom..?" sa hon. Korkade, ansvarslösa barnrumpa!<br><br>Nu ser vi att hela Uppsala fått ännu strängare regler, men de enda som lyder dem är - vi, de äldre!! De yngre sprider glatt smittan vidare och vi måste bära oket.<br><br>Men - de är också dödliga..			

32	2020031 8	Samhällsekonomin, risk att många blir arbetslösa, hushållen är högt skuldsatta. Vad händer med priserna på bostadsmarknaden mm. Många kanske får sälja med stora förluster och sedan dras med blancokrediter med hög ränta i många år	Inte mycket, tänker att vi trots allt klarar detta. Men behöver inte vara så orolig för egen del och det gör det ju lite lättare..	Ja, undviker större folksamlingar, tvättar händerna oftare och mer noga, håller längre distans till andra, tar inte gärna i hand eller kramas. Går dock fortfarande ut och äter på luncherna men undviker buffémat och väljer mer noggrant vart jag går. Tänker också mycket mer på vilka restauranger jag helst vill stötta.	Hej! Mest rädd är jag för att det kan få mer långsiktiga konsekvenser på människors psykiska hälsa. T ex ensamhet bland främst äldre, men även bland yngre. Svårare att komma ut på arbetsmarknaden påverkar också den psykiska hälsan. Ja, det påverkar oss alla på olika sätt.		kvinna	1966
34	2020031 8	Att det ska drabba de som blir så sjuka att de behöver sjukhusvård! Att det drar ut för långt i tid, så att ekonomin kraschar för små o stora företagare.	Jag har blivit mer försiktig och eftertänksam, försöker tänka stort, vad kan jag göra för att underlätta eller hjälpa till i det läge som råder?!	Följer rådet om social distans - närhet, men veckohandlar o vistas ute, försöker leva så nära vanligt som möjligt. Mycket fler o noggrannare handtvätt tvål o vatten hemma o handsprit i bilen o handväskan!		Rädslan är nog fortfarande densamma, att bli sjuk o smitta våra gamla föräldrar o andra!<br>Naturligtvis också att bli så sjuk så man behöver belasta sjukvården!	kvinna	1954
38	2020031 8	Det är ovissheten som är värst. Gör vi rätt eller fel? Hur sjuk kommer jag att bli? Fixar jag hostan?	En overklighetskänsl a. En lustig känsla i magen.	Minskat på sociala kontakter. Träffar inte mina barnbarn. Handlar inte lika ofta.	Jag är mest rädd för människors arrogans och tro på att det inte ska drabba dom.		kvinna	1960

40	2020031 8	Att det tar så lång tid innan allt fungerar normalt igen. Tycker synd om alla som har butik, restauranger eller som jobbar i dessa branscher de har några få eller inga kunder alls.	Är inte rädd för att handla mat men igår på seniordagen var det betydligt mindre kunder än en normal tisdag.	Tycker att det pratas för mycket om corona information är viktigt och det behöver ta del av men det får inte så så långt så människor skräms och blir ännu mer oroliga för vad som ska hända. Det har påverkat alla vi hockeyfans som skulle gått på kvartsfinal matcher nu i slutet av mars men så blir det inte i år. Sen skulle jag deltaga i arrangemang i mitten av april som jag ännu inte vet om det blir av.	Tiden det tar innan vi får ett vaccin så att vi kan börja leva normalt igen, jag trodde det skulle avta under sensommaren och sen plana ut under höst och vinter, men istället så ökar det igen. Börjar bli trött på allt vad corona heter, men håller avstånd när jag är ute och handlar. I mitt bostadsområde finns många gångstigar så det är lätt att vara ute och få en promenad varje dag utan att möta många människor. Längtar efter den dagen när jag kan inta min plats i Catena Arena igen.		Kvinna	1953
58	2020031 8	Jag är rädd för att ett ensidigt fokus på att begränsa smittspridning kommer ge allvarliga och långtgående konsekvenser för samhället vilket medför mer död och eländet än viruset någonsin kunnat.	Blir förbannad växlat med likgiltighet och en vilja att kämpa emot det jag ser som problemet	Nej. Absolut ingenting. Påtvingats en del förändringar på grund av andras reaktioner dock.	Mina största rädslor är att det används som förevändning för inskränkningar av friheter, långtgående omdaningar av samhällsstrukturen samt att de gigantiska lån och finansiella beslut som tas kommer rasera vår levnadsstandard och leda till långt värre konsekvenser än sjukdomen i sig. Sjukdomen i sig har jag ingen rädsla för.		Man	1987

71	20200318	Att gamla som är i riskzonen ska smittas och bli sjuka. Tänker mycket på min morfar. Jag är också rädd för att skolor ska stänga eftersom jag tror det kommer skapa kaos för oss som jobbar inom sjukvården. Många behöver vara hemma med sina barn och då blir det ännu mer att göra för oss som är kvar och jobbar.	Det skapar oro. Men utöver min oro känner jag inte att jag påverkas jättemycket.	Tvättar mina händer otroligt mycket oftare. Vill inte vara för nära någon som låter sjuk på pendeltåget. Träffar inte kompisar i samma utsträckning. Tankarna är även helt annorlunda än normalt.		Jag känner mig faktiskt inte så rädd för att själv drabbas av covid-19. Jag är mer rädd för att någon av mina nära och kära ska drabbas och bli allvarligt sjuk. Jag är rädd för att det kommer bli värre och värre inom sjukvården och att vårdpersonalen inte kommer klara av det höga trycket. Jag är rädd för att stor del av kollegor ska bli sjuka vilket gör att det blir ohanterligt för de som är kvar och jobbar. Finns mycket som känns skrämmande i hela samhället, har svårt att sätta ord på allt. Längtar tills allt är som vanligt igen...	Kvinna	1992

74	20200318	Rädd för att vara smittspridare till någon i riskgruppen. Spritar händerna i absurdum i butiken där jag jobbar. Rädd för ekonomisk kollaps. Rädd för ovissheten koppad till mörkertalet av smittade.	Svårt att fatta beslut. Stress. Placebo-symptom uppstår. Blir trött och isolerar mig	Mycket förändrad. Avstår från extrapass på jobbet, går inte ut i onödan. Tar inte på allmänna ytor. Spritar händerna om jag ändå gör det. Ställer in träningspass. Ställer in träffar med vänner. Ställt in att åka till en begravning.		Jag är mest rädd för att omedvetet råka sprida vidare smittan och rädd för att söka vård orelaterad till covid-19 (vill inte bidra till den redan rådande höga belastningen på vården). Rädslan till att omedvetet råka sprida vidare smittan ger upphov till konstant orohetskänsla när man befinner sig utanför hemmet då många inte respekterar att hålla avstånd. Försöker vara extra noggrann med avstånd till äldre och blir stressad på deras vägnar när jag ser att andra inte respekterar dem.	Kvinna	1989

75	20200318	Jag tycker att ovissheten om hur allvarligt Corona faktiskt är och vilka konsekvenser det kommer få i samhället/världsekonomin etc är det jag är mest rädd för.	Det som händer är främst att jag kollar mycket på nyheterna, läser på nätet och sociala medier, diskuterar med vänner och familj om hela situationen. Jag har också blivit mer försiktig i vardagen för att inte bli smittad eller sprida en smitta.	Ja! Jag har handlat lite mer mat än vanligt via nätet, undviker att åka tunnelbana i rusningen, har inte gått ut på restaurang och såklart inte heller rest utomlands.	Det jag känner mig helt klart mest rädd för just nu är hur länge den här pandemin kommer hålla i sig och hur länge den kommer ha ett grepp om världen. I början hade man en förhoppning om att det sakteligen skulle "dö ut" och in bli som vanligt igen men nu känns det allt annat än så och länder stänger ner igen. Jag tycker det känns läskigt att tänka att det kanske 2-5 år framåt kommer vara snack om Corona och inte bli som vanligt igen. Sen är jag rädd för hur lång tid det kommer ta att få fram vaccin för att sen kunna vaccinera en så stor andel människor i hela världen.		Kvinna	1988

83	2020031<br>8	För att ekonomin ska kollapsa p.g.a marknadens panik. Är du inte så rädd för corona. Det är som en svårare influensa	Jag blir nedstämd och sammanbiten	Använder handsprit. Håller socialt avstånd om det inte är viktigt. Är uppmärksam på om någon nyser och flyttar då på mig	Jag är rädd för världsekonomin går i botten vilket leder till svält och fattigdom och Kina köper alla företag som är på väg mot konkurs.<br><br>Jag är rädd att detta kommer att pågå under flera år och vi måste ta vaccin som jag är rädd för		man	1955

	84	20200318	Jag är mest rädd för att min mamma (75 år) eller någon av mina svärföräldrar (80+) eller äldre vänner ska drabbas av viruset och få lunginflammationen. De är alla friska och hälsosamma så jag tror att de kommer klara sig bra, men jag är också beredd på att någon kan råka illa ut. Jag är också bekymrad över att många inte tar myndigheternas rekommendationer på allvar så att vi får en mycket värre topp på smittan med efterföljande konsekvenser för sjukvården och samhället i stort. Våra system är bräckligare än många tror. Vi kommer att klara oss genom det här men det är synd om vi gör saken värre genom att bete oss egoistiskt.	Jag är inte särskilt rädd just nu (2 på en 10-gradig skala). När jag blir rädd på riktigt så blir jag nedstämd och kan ibland fokusera för mycket på problemet och förstora det. När läget är akut på riktigt (eller människor omkring mig får panik) så agerar jag oftast lugnt och metodiskt och håller huvudet kallt.	Inte på grund av rädsla. Jag undviker att gå på affärer för att minska risken att jag sprider smittan till någon. Istället rullar jag beredskapslagren och försöker handla högst en gång i veckan. Jag jobbar också hemifrån och ringer min mamma (som är änka) och håller kontakten med vänner mer än vanligt för att uppväga den sociala isoleringen för mig och dem. Jag försöker lugna mina kollegor och de som jobbar åt mig i teamet på jobbet.	Jag är inte särskilt rädd för egen del. Min mor, som är över 70 år gammal, har så att säga ”kommit in i lunken” och har inrättat sitt liv så att hon kan undvika att bli smittad utan att bli helt isolerad och instängd. Vi har också kommit en bit med vårdmetoderna för de som drabbas av viruset så att fler av de svårast drabbade förhoppningsvis kan räddas. Jag tror också att vi har kapacitet och uthållighet kvar i Sverige att skärpa till oss och följa rekommendationerna från Folkhälsomyndigheten och det regionala smittskyddet. Vi har också råd att införa ytterligare restriktioner utan att riskera vår ekonomi långsiktigt.<br>Det som jag är mest rädd för just nu är faktiskt att situationen i omvärlden radikaliseras ytterligare och att de reaktionära och auktoritära krafterna runt om i världen lyckas flytta fram sina positioner och i vissa fall också lyckas permanenta sitt maktinnehav.<br>Viruspandemin skapar oro och många verkar försöka ta chansen att sprida propaganda som eroderar tilltron till samhället och vår gemensamma kraft att		man	1970	

					hantera situationer som denna. Jag tror tyvärr att om republikanerna lyckas behålla och permanenta sin makt och presidentmakten i USA efter valet (oavsett faktisk valutgång) så kan problemen med polarisering och radikalisering eskalera snabbare än vi klarar av att hantera dem. Kombinationen av pandemi och högerpopulism/auktoritarianism skrämmer mig.			

88	20200318	Främst är jag rädd för vad som kommer hända i efterdyningarna. Många som blivit av med sin inkomst vilket kommer öka trycket hos redan ansträngda kommuner samt en arbetsförmedling i total oreda. Finns också väldigt många som kommer råka illa ut hemma, våld i nära relation eller barn som blir utsatta utan att det kan upptäckas i samma utsträckning av övriga samhället. Är också rädd för hur vissa samhällsfunktioner kommer kunna upprätthållas under pågående utbrott. Samt en del människors beteende skrämmer mig också.	Jag jobbar själv inom kommunen och på enheten jag jobbar är det redan svårt att hinna med. Jag hoppas att det kommer mer stöd från staten så att människor med osäkra anställningar kan få mer säkerhet. Jag kan känna en hopplöshet. Men så ser man guldkorn, att en del kommer snabbutbildas till att kunna underlätta för vården och det gör att jag ser hopp och känner mig glad. Jag försöker vara noggrann att tänka igenom var min rädsla ligger för att inte själv vara ett problem, eller vad man ska säga. Ett problem med min rädsla är att jag suger i mig allt som skrivs om Corona, när man egentligen ibland kanske borde stänga av.	Jag har varit hemma med någon form av influensa-variant tillsammans med min familj. Jag är ute i naturen och försöker kurera mig och inte komma tillbaka för tidigt. Är inte orolig för egen del att bli smittad, däremot vill jag inte riskera att sprida smitta. Jag försöker vara extra noggrann med att få min treåring att tvätta händerna. När jag är frisk kommer jag hålla mig hemma mer än vad jag tidigare gjort, även om jag är lite trött på att vara hemma så mycket vid det här laget.	Kommer inte ihåg vad jag svarade sist men tror att det är ganska mycket samma. Har inte varit så rädd för att själv bli smittad men är rädd att föra vidare smitta. Är nog dock mer rädd för att själv bli smittad nu än jag var innan. Jag flyttade till Kalifornien i juni och vi har haft stora problem först med värmebölja och sedan med skogsbränder. Det tog udden lite av pandemin för helt plötsligt var vi tvungna att vara beredda på att evakuera. Men det gjorde också att jag blev mer rädd för att bli smittad. Jag har astma som jag i vanliga fall inte märker av men när luftkvaliteten börjar gå över gränsvärdena märker jag av den. Vi bor inte i ett så tätt hus och de luftrenare vi fick tag på räckte inte för att hålla kvaliteten bra i hela huset. Det var flera gånger det var svårt att andas inomhus och ibland när jag gick och la mig hoppades jag att det bara var luftkvaliteten och inte covid också. Var extra rädd för att få covid under den perioden eftersom mina lungor redan kämpade. Förutom den mer ”akuta” rädslan så är jag rädd för att vi inte ska få något ”normalläge” när vi bor här samt att vi inte ska		Kvinna	1987

					kunna åka hem för att hälsa på eller få besök. Det har blivit en ganska ensam tillvaro eftersom vi inte träffar nya människor mer än ytligt. Är rädd för de långtgående konsekvenserna i människors psykiska mående. Här är också skolorna stängda så barnen har distansundervisning, jag är rädd för vad det kommer göra med den generationen som blir så isolerade. Vår son är i förskoleåldern och går på förskola 3 dagar i veckan, men har bekanta med barn i distansundervisning som verkligen kämpar.			

97	2020031<br>8	Att folk inte förstår faran med att nysa o hosta rakt ut o att de går ut o är sociala då de är lite småsjuka , trots vad myndigheterna sagt - att folk inte fattar vad som sägs - och hur fort viruset sprids o hur farligt det är gör riskgrupperna	Ibland märker jag att jag blir lättare arg , jag går in i ’ fight’ läge lättare än annars .	Jag har hållt mitt barn på 18 år hemma från gymnasiet trots att det varit skolplikt - i 14 dagar nu - för jag ansåg att FHM hade fel med att vänta med vissa restriktioner - anser det fortfarande - igår togs beslutet på att gymnasielever kan läsa hemma och det kändes väldigt bra för mig . Så jag slapp ha skuldkänslor gör att jag tagit det beslutet själv - nu blir min son som ’ alla andra’. Jag har en kronisk sjukdom i nervsystemet , samt astma - så därför vet jag att det finns risk att jag skulle behöva IVA om detta viruset hår ner i mina lungor o jag förstår att det finns inte plats för alla på IVA snart - o det innebär att läkare får välja vilka de ska satsa på ... tufft.	Minns ej vad jag svarade förra gången :)<br><br>Jag är inte särskilt rädd nu iaf , jag är försiktig o gör som FHM vill .<br><br>Jag har respekt för viruset , har inte fått det , ännu ...<br><br>Eftersom jag har nedsatt immunförsvar och en jobbig astma också , så skulle det absolut vara jobbigt om jag fick covid .<br><br>Men jag tror det blir oundvikligt , till slut , och har nog fått en acceptans för det , därav mindre rädsla .<br><br>Jag tar inte i hand och jag kramar inte om någon utanför familjen :)		Kvinna	1961
98	2020031<br>8	Kaos i sjukvården.	Ingenting förutom att följ råden och ligga lågt.	Jag är inte rädd för att bli smittad utan försöker vara försiktig för kollektivets skull.	Mest rädd för nu...<br>Att det skulle dröja mycket länge innan det finns vaccin!		Kvinna	1956

109	20200318	Jag känner mest en oro för all konflikt som uppstår i samband med detta. Alla slitningar mellan människor som tycker att allt hanteras helt rätt eller helt fel. All energi som läggs på att vara osams istället för att bara lugnt lyssna på de källor myndigheterna hänvisar till och göra som de säger. Jag oroar mig också mycket för vad som händer med miljön. Det är min stora sorg och oro alltid - just nu minskar utsläpp på grund av att människor sätts i karantän, sjukskriver sig och så vidare, men vad kommer att hända när allt det här är över? Tar samhället chansen att fortsätta omställningen mot ett miljövänligare leverne, nu när vi ändå är lite på väg genom åtgärderna kring corona, eller blir det värsta bakslaget, med stöd till industrin och flyget, värsta uppsvinget för resor och konsumtion, bara för att människor vill ta igen det de tycker att	Som sagt, min rädsla och oro och sorg gäller mest miljön. Och det yttrar sig mest som sorg.	Jag har en lätt förkylning nu. Den skulle inte ha fått mig att stanna hemma i vanliga fall, det är bara lite snuva och värk i kroppen. Men nu håller jag mig inne och avbokar allt. Det känns märkligt, men på sätt och vis också lite skönt.		Förutom oron att någon när och kär eller jag själv ska bli svårt sjuk och kanske dö, så handlar min oro mest om miljön.<br>Jag tror att många kommer att se minskat resande och minskad konsumtion bara från den negativa sidan, som en förlust, inskränkningar och det hemska och svåra i att företag går under och människor blir arbetslösa – och därmed kommer det att bli ett enormt motstånd mot att göra saker som är bra för miljön, som att låta bli att flyga eller konsumera.	kvinna	1968

		de gått miste om? Vad gäller smittan så är jag mest rädd att smitta andra. Jag jobbar med ideella grupper med många äldre och hoppas innerligt att jag inte fört någon smitta med mig som utsätter någon för fara eller belastar sjukvården på sikt!						

113	20200318	Folks panik, man fattar korkade beslut utan att tänka. Tydligt att det finns filter bubblor och att sociala medier spär på detta.	Nu blir jag bara irriterad, när jag blir rädd på riktigt blir jag mest passiv/observerande	Nej	Just nu, USA valet samt vår oförmåga att lägga undan maktsökande för att samarbeta kring de riktiga problemen (miljö)		Man	1961
115	20200318	Jag är inte rädd för något just nu. Jag väljer inte heller att vara orolig innan något har hänt vad gäller viruset för egen del.	Jag blir sällan/aldrig rädd för något som inte har hänt.	Vad gäller viruset så har jag en ökad uppmärksamhet på om folk i min omgivning är förkylda och undviker större folksamlingar, jobbar hemma när så är möjligt o s v.	Jag är mest rädd för att Donald Trump ska återväljas som president i USA. Det är den enskilda sak som har mest påverkan på diverse globala miljöfrågor. Men ordet rädd är i starkaste laget, oroad är mera relevant för vad jag känner.		Man	1956

119	20200319	Jag är mest rädd för att det redan spända politiska läget i världen ska eskalera och förvärra det hela. Att människors rädsla ska leda till konflikt istället för att se det som att vi alla sitter i samma båt. Vi är så lyckligt lottade i den här krisen att vi har möjlighet att luta oss mot vården och en regering som ändå ser till människan. I kina sköt de ihjäl husdjur och svetsade igen dörrar. Vi har det bättre men jag är rädd att människors rädsla kommer vara det som stjälper oss i "ensam är stark" landet Sverige.	Det ligger som en molande värk strax under solar plexus och som en tunn slöja av brännande tårar så fort jag stannar upp och reflekterar över vad det är vi möter just nu.	Jag är inte rädd att i sig bli smittad, men jag jobbar stenhårt på att inte smitta vidare. Jag jobbar i vården som samordnare och jag måste se till att jag och mina kollegor håller. Att vi är friska och orkar stå emot när toppen kommer.	Är jag rädd?<br>Ja, är det korta svaret. Det enkla svaret. Det mest koncisa.<br>Men också det svar som allra mest saknar nyanser.<br>Vad är jag rädd för?<br>Jag är rädd för att bli sjuk, och tappa tid med mitt barn.<br>Jag är rädd för att dö ifrån mitt barn.<br>För om jag är död finns jag inte där när hon behöver mig.<br>Det skrämmer mig att inte få vara där när hon lär sig cykla, eller simma. Att inte få se henne skriva sitt eget namn första gången, eller få krama henne första gången hon upplever hjärtesorg.<br>Du frågade mig om jag är rädd. Ja, snyftade jag till svar utan personalprovtagningen.<br>Jag har haft två insjuknade kollegor och där stod jag. Täppt i näsan, ont i halsen.<br>Vem ska berätta för henne vem jag var om jag inte finns?<br>Vem ska berätta för henne att jag dansade när jag lagade mat, att jag levde på clementiner när jag väntade henne och att jag varje kväll strök henne över håret och viskade: "Mumpan. Min kärlek. den jag älskar mest."<br>Vem ska berätta för henne		Kvinna	1984

					att jag klättrade 26 meter ner i en grotta i Mexiko, att jag en gång pratat om sex på radio och att jag allra helst dricker kaffe klockan 6 på morgonen vid ett gläntat fönster?<br>Är jag rädd?<br>Ja.<br>Är jag smittad?<br>Nej.			

120	20200319	Jag har alltid varit en hypokondriker och haft bacillskräck men märkligt nog är jag inte ett dugg rädd nu för själva viruset. Däremot är jag rädd för följderna det får för samhället - massarbetslöshet och fattigdom	Kan känna en depression men ingen rädsla	Nej, inte mycket mer än att jag spritar händerna ofta	Jag är fortfarande inte rädd för viruset. Däremot kan jag känna ett obehag när jag läser om restriktionerna i till exempel Italien, Storbritannien och Spanien och tänka att länderna kommer att kollapsa ekonomiskt.<br>Jag är också rädd för att många kommer bli psykiskt sjuka.		Kvinna	1956
123	20200319	Att jag ska bli akut sjuk och inte få hjälp.	Allmän dödsångest.	Håller avstånd till människor och undviker folksamlingar.	Ja risken att smittas har ju ökat rejält så rädslan att bli svårt sjuk/dö har ökat. Som äldre astmatiker är prognosen inte särskilt ljus.		Kvinna	1959
126	20200319	Att mina barn+barnbarn blir sjuka. Inte för egen del.		Inte ändrat så mycket, lever ensam som sjukpensionär.		Är rädd för att mina nära o kära ska drabbas, inte för egen del.	Kvinna	1961
127	20200319	Ekonomisk kollaps och att bli arbetslös - eftersom jag arbetar för ett privat företag, ett litet sådant.	Ångest, uppgivenhet - och en känsla av att det får bära eller brista.	Har digital kontakt med nära och kära. Annars är det främst mer noggrann hygien som gäller.		Jag är mest rädd för att vår välfärd ska monteras ner till intet. Att det blir en dominoeffekt av det här. Att fler människor hamnar utanför samhället. Att vi bli mer trångsynta och rädda för varandra.	Kvinna	1987

128	20200319	Jag är rädd för de följder som hela den situation världen just nu befinner sig i, kommer att få. Hur många företag går i konkurs? Hur många kommer att förlora sina jobb? Kommer vi att få se en stor ökning av depressioner och självmord? Hur ska det gå för hela kulturbranschen? Ännu mindre pengar framöver. Jag är rädd för att de negativa effekter all nedstängning av olika verksamheter får är betydligt allvarligare än följden av viruset i sig. Jag är inte orolig för att själv bli sjuk, men inser att det kan bli farligt om min gamla pappa, eller vänner som är sjuka får viruset.	Jag får en känsla av tryck över strupen och halsen. Känner mig allmänt nedstämd och orolig. Svårt att koncentrera mig på andra saker.	Egentligen inte särskilt mycket. Cyklar till jobbet, så det går ju bra att fortsätta med. Kanske äter mer sällan ute på lunchen. Och ja - som alla andra tvättar jag händerna betydligt oftare och noggrannare än vanligt.	Att ingenting någonsin ska bli som förut… Världen som vi kände den, med för mig stort socialt umgänge, kulturella upplevelser, sammanhang där det riskerar att bli trångt, resor till när och fjärran. Jag är väldigt fysisk av mig och saknar dagliga kramar från goa kollegor och vänner. Har jobbat hemifrån till stor del sedan mitten av mars. Tack och lov för att jag har min underbara sambo! - All denna distansiering. Det känns så onaturligt för mig. Känslan av att inte riktigt vara välkommen, vare sig det är hos vänner eller i andra länder. - Är fortfarande inte rädd för att själv bli sjuk. Mer för följderna denna pandemi får för hela världen. Känner mig däremot väldigt dämpad och låg. Är så trött på Corona!		Kvinna	1967

129	20200319	Att förlora nära och kära. Behövs inte förklaras.	Får en stor ovisshetskänsla. Får också en otäck känsla av ett hot man inte själv kan påverka som dessutom inkräktar på ens liv. Psykiskt påfrestande.	Ja, till viss del. Mycket med hänsyn till andra. Riskgrupper och gamla i min närhet. Undviker mycket folk. Jobbar hemma. Jag har ju möjlighet att göra så för att hjälpa till och inte bidra till smittspridningen. Smittad blir man nog förr eller senare själv. Lite oro för det med. Åker gärna ut i naturen där man kan koppla av en stund. I och för sig har jag alltid gjort det men nu betyder det ännu mer. Man får distans till eländet.		Jag vill inte ha viruset, är också rädd att det ska drabba familj och vänner då man aldrig vet hur det kommer att bli. Sen är man rädd att bli av med jobbet då det verkar bli en långdragen pandemi.<br>Var permitterad i våras och det var nog på håret att man fick stanna. Nu fick vi en höst vi inte hade hoppats på och<br>arbetsgivare ser än en gång över sina resurser.	Kvinna	1967
139	20200319	Att bli långvarigt ensamen.	Jag blir orolig och ledsen.	Tvättar händerna noggrannare.		Jag är mest rädd för ensamheten, att min mamma ska drabbas och att pandemin aldrig ska ta slut.	Kvinna	1974
148	20200320	Ovissheten! Vad ska man tro? Blir det en sån stor epedemi som en del tror eller är det media som har gjort situationen hysterisk.	Inget! Lite orolig för familjemedlemmar med astma.	Har inte ändrat något mer än att tvätta händerna regelbundet. Håller lite mer avstånd till andra människor.	Jag är inte speciellt rädd för något.<br>Lite fundersam inför hur det ska utvecklas inom närmsta månaderna. Jag är 65år och lever precis som vanligt både med jobb och fritid. Tänker dock på viktiga saker typ handhygienen m.m		Kvinna	1955

151	20200320	Samhällskonsekvenser, individers kanske ännu mer begränsade möjligheter få "rättigheter"/service, försäkringsskydd (samhällskontraktet) utifrån rätt/lag, inte praxis eller den starkes rätt!		Taggar utåt...	Personligen är min/mina största rädslor personligen inte nära kopplade till corona!<br>Samhällelig förändring som följt/följer som konsekvens är både skrämmande & hoppväckande som de flesta förändringar:)		Man	1964
152	20200320	Att det inte skall finnas vård om jag så behöver det sekundära problem är att mitt företag går i konkurs	Oroligt. Samtidigt som jag är upprörd och förbannad över den senfärdighet som regeringen uppvisar var gång det blir en kris av något slag. Vi har en så kallad som ändrar sina tyckande för varje dag som han uppträder! Vilket blir helt omöjligt att kunna finna ett personligt sätt att kunna hantera denna kris/epidemin så sammanfattnings vis JAG har inget förtroende för vare sig regeringen eller deras så kallade EXPERTER	Har inte råd att ändra mitt levnadsmönster	Jag är fortfarande nervös/rädd att bli drabbad av Covid-19 då jag är riskgrupp. Samt att min granne dog i Corona efter 70 dagar i respirator.		Man	1968

158	20200320	Att jag eller nära ska bli riktigt sjuka och kanske inte klara sig då det inte finns botemedel eller vaccin i nuläget. Eller att vården inte klarar av situationen och att när man väl behöver hjälp så finns det inte plats.	Ångest och konstant oro.	Social isolering (fysisk) och påverkar även mina barn att de får ändra deras sociala kontakter och hygien påminnelser	Det jag mest är rädd för nu är att pandemin kommer vara kvar under överskådlig tid och påverka vårt samhälle och världen på ett fortsatt negativt sätt. Jag är nog rädd att det kommer att bli mycket värre innan det här vänder. Om det vänder. Längtar se efter ett ljus i tunneln.		Kvinna	1970
162	20200320	Att det ska bli karantän för alla, att Sverige stänger ner.	Jag blir ledsen och tänker mkt på hur det eventuellt blir.	Mina arbetsuppgifter har ändrats eftersom alla verksamhet är inställd tillsvidare. Jag använder latexhandskar när jag handlar. Håller avstånd till andra människor och håller mig hemma på fritiden.	Jag är mest rädd för att bli smittad av Coronaviruset på arbetet. Mina kollegor stannar inte hemma när de har symtom. De håller inte avstånd och tvättar inte händerna. Jag är i riskgrupp på grund av medicinering och är orolig för att bli väldigt sjuk och få bestående skador på lungorna och därmed förkortad livslängd.<br>Jag är också väldigt orolig för min gamla mamma, 77 år, som bor själv 15 mil från mig. Hon envisas med att handla själv trots att kyrkans personal erbjudit sig.		Kvinna	1967

	16<br>4	2020032<br>0	Är rädd för att behöva intensivvård när det är som värst..är rädd för den ekonomiska krisen i svallvågorna efter corona.	Blir snabbandad..lite panikslagen av att inte kunna göra något åt det... gråter mycket i smyg	Tvättar händer...undviker folksamlingar...försöker tänka sunt	Ja, vad är jag rädd för nu.... Den akuta "jag vill inte dö-känslan" har lugnat sig. Är nu mer arg på corona. Jag har inte kunnat krama min mamma sedan i mars, jag har inte fått vara hos henne på sjukhuset när hon behövde mig. Hon fick ligga där ensam och rädd! Jag är rädd för att detta aldrig blir bra igen....rädd för att samhällets ekonomi inte går att återhämta!		Kvinna	1965	

	170	20200320	Att nära och kära med underliggande sjukdomar och hög ålder blir smittade av Corona och blir allvarligt sjuka. Att själv i förlängningen bli utförsäkrad pga ekonomisk nedgång. Arbetslös legitimerad lärare sedan jan. Har viss funktionsnedsättning men har ändå kallt räknat med att hitta nytt arbete senast till hösten 2020. Det känns inte alls lika säkert längre och jag hamnar mellan stolarna i vården redan nu.	Jag distansierar mig mentalt lite för mycket, blir handlingsförlamad vad gäller mig själv och min egen situation. Kan hjälpa andra bra och lugnt. Läser in mig på viruset. Kunskap lugnar mig.	Ja. I början mest rädd för egen del. Nu mest för att bidra till att smittan inte sprids i samhället. Pga arbetslös och deprimerad redan i självkarantän i princip. Ställde in spadag med mor, moster och kusin, nekat fika pga sambo har symptom, handlat 1g/vecka. Planerat med varor hemma för att klara 14 dagar utan handling.	Just nu är jag mest rädd för min mor som testade positivt för Covid19 i början av sommaren. Hon har inte behövt sjukhusvård men hon blir inte frisk. Hon blir snarare stadigt sämre. Hon är orolig och rädd för att smitta andra fastän hon enligt läkarna inte ska vara smittsam längre. Det har inte känts som att vården tagit hennes symptom på allvar men hon har först nu efter snart 4 månader fått en hjärtutredning och träffat regionens covidansvarige läkare.<br>Det har blivit allt svårare för mina 70+föräldrar att leva i stort sett isolerade från andra. Nu när rekommendationerna har mjukats upp så är jag istället orolig för att min far inte klarar av att hålla avstånd, tvätta händerna och inte nudda ansiktet mm. Han glömmer bort viruset och tar det inte på allvar trots att han är utbildad sjuksköterska.<br>Jag är också orolig för hur många yngre vuxna inte tar sitt ansvar och blev faktiskt chockad av hur studentnationerna i Uppsala (där jag en gång själv studerat) verkar ha kört på som vanligt med gasquer etc. i sina gamla, trånga,		Kvinna	1972	

					dåligt ventilerade lokaler.<br><br>Själv är jag arbetslös lärare och är frustrerad över hur många skolor fortfarande inte låter lärare arbeta hemifrån när de inte har undervisning. Lärare tvingas likafullt ut i kollektivtrafiken för stora gemensamma föreläsningar etc. när eleverna har lov.<br><br>Det gör att jag oroas för utvecklingen under hösten och vintern. Jag behövde själv två operationer i våras och är tacksam för att det fanns personal till dem. Nu är det synnerligen oroande att vårdpersonalen som jobbat extra hårt sedan mars inte får någon lättnad i och med att covidfallen ökar. Vi är därför sämre rustade nu om smittan exploderar. Utan kompetent personal kan vi bara erbjuda sängar på sjukhusen.			

174	20200321	Jag är inte rädd för att få viruset men det jag är rädd för är att jag ska smitta någon som inte klarar av det. Jag jobbar även inom vården och är rädd för vad jag kommer få se om några veckor. Om man måste börja prioritera vilka som ska få leva och inte. Det skrämmer mig.	Jag sitter och läser på om det och försöker hålla mig uppdaterad.	Jag går inte ut lika ofta och eftersom skolan även är inställd så blir det mycket tid hemma.		Det jag är mest rädd för just nu är att pandemin ska hålla på i flera år framöver. Att det inte kommer gå tillbaka till det ”vanliga” och att viruset muterar sig och blir värre än innan. Jag är rädd för att gå in i en depression och just nu i dessa tider är det svårt att ta sig ur det. Något som jag även är rädd över är att någon nära ska gå bort nu då man försökt att inte träffats så ofta. Det är mycket rädslor och att inte veta ett slutdatum är riktigt jobbigt.	Kvinna	1997
179	20200321	Att vi förstör vårt samhälle. River infrastruktur, viktiga samhällsfunktioner, välfärd och människors liv med en panikartad situation pga av ett virus som troligen inte är mycket farligare än en svår säsongsinfluensa	Inte mycket. Fortsätter min vardag som vanligt i största möjliga mån	Nej	Mest rädd för att vi river sönder en samhällsstruktur som vi svårligen kan bygga upp igen. Människors navia teorier om skydd mot virus, agerande byggt på falsarier, i bästa fall på de förut nämnda naiva teorierna, ställer till mer och dödar fler än viruset i sig. Jag är inte rädd för viruset, jag är rädd för människans irrationella, ologiska beteende. Ofta byggt på dumhet och okunskap.		Man	1964

18<br>1	2020032<br>1	Jag är mest rädd för att jag eller någon i min familj ska vara tvungen att åka in akut till sjukhuset av någon anledning (behöver inte vara covid-19) och då inte få hjälp i tid för att sjukvården redan är överbelastad trots att vi inte ens nått kulmen.	Jag blir mindre social och grubblar mer. Jag undviker att träffa folk. Har däremot börjat ringa dem jag inte ringer så ofta.	Jag handlar mat online. Går inte på köpcentrum eller andra ställen med mycket folk. Tvättar händerna väldigt ofta. Vi är mycket hemma eller i naturen.	Mest rädd just nu är jag för att göra en felbedömning av smittoläget så min familj och jag blir smittade. Det är svårt att veta hur allvarligt läget faktiskt är. Det finns en jargong som är "ja, ja får jag det så får jag det men det är inget att vara så himla orolig för" och försiktiga människor ses på som hysteriska och överdrivna samtidigt som smittan ökar snabbt i samhället.		Kvinna	1975

182	20200321	Att människor ska bli rasister, att gränserna ska fortsätta vara stängda, att de som fastnat i flyktingläger dör. Det är som ett eko av Förintelsen. Men den här gången utan gas.	Jag blir apatisk. Eftersom det inte hjälper att gråta, eller skriva petitioner, eftersom ingen lyssnar, vad ska jag göra? Jag berättar, jag gör berättelser, jag försöker väcka empatin hos de vars empati har slocknat.	Ja, och det är vidrigt. Jag tvättar mig hela tiden, tvättar dörrhandtag, har papper med, försöker undvika att trycka på knappen på bussen, tar en annan väg på jobbet än jag brukar, handikappingången har en automatisk dörr, så att jag slipper ta i dörrhandtag.	Jag är mest rädd för den främlingsfientlighet, den rasism, som växer upp i skuggan av pandemin. På något sätt får man det alltid till att det är "invandrarnas fel". KD trodde att människor från Somalia inte förstod, som om de aldrig någonsin skulle titta på tv eller läsa tidningar. Och om de nu inte förstår svenska, så kan man läsa tidningar och få nyheter på nätet, på sitt eget språk. De visste, men tvingades köra turister som varit utomlands och tagit med sig smittan. - Covid-19 drabbar människor som inte kan arbeta på distans, de som kör taxi och sitter i en liten bil, nära passagerarna. Det drabbar människor inom vården, som måste ta i sina vårdtagare, vara nära ansiktet, det finns inga andra val. Då får invandrare skulden igen. Både för att de blir smittade och för att de sprider smittan vidare. - Okunniga människor, långt bort från vården, påstår att invandrare skulle vara dumma i huvudet och att smittan skulle minska om fler svenskar arbetar inom vården. -Ytterligare ett skäl som får rasismen att blomstra är stängningen av gränserna. Det är		Kvinna	1961

					nödvändigt, men för den som flyr finns inga val, men måste korsa en gräns eller flera. Vi sätter inte in vita bussar, båtar, flyg, för att rädda människor från att drunkna i Medelhavet, eller bli skjutna av gränsvakter. Och vi kan luta oss mot att gränserna är stängda på grund av Corona. Det är så vidrigt. Den lilla medmänsklighet vi hade, försvinner. Det är det jag är mest rädd för.			

	18<br>3	2020032<br>1	Ekonomin. Vilka (små-)företag finns kvar, hur mycket kultur kan överleva, hur kommer sjukvård och övrig omvårdnad bli mm? Rädd för hur vissa drabbas av (orationell) panik och beter sig "knäppt" o roffar åt sig medicin mm. Rädd att en positiv människosyn försvinner ännu mer..	Kallsvettas, ont i magen, ångest, sorg	Eftersom jag har medicinska problem, bl a med min andning, så har jag valt självvald karaktän. (Min vuxna son som bor hemma, jobbar däremot och det uppmuntrar jag)<br>Träffar inte mitt 1,5 åriga barnbarn, min andre son och hans fru. Träffar ingen alls utom sonen som bor hemma<br>Tvättar händerna oftare	Det jag mest är rädd för nu är : att det aldrig tar slut. Man hör om muterade virus, ny stor "våg" av smittade osv<br>Har varit i självvald isolering sedan i mars, träffat någon få på utefika med avstånd, men nu funkar det ju inte med utefikor längre. Undrar om jag någonsin kan krama och leka med mitt barnbarn, och umgås normalt med nära och kära igen<br>Just nu känns allt rätt svart!		Kvinna	1971

18<br>4	2020032<br>1	Att våra barn kommer att få en tuff framtid. Inte bara pga covid19, utan vi vuxna och våra förfäder har fördärvat planeten nåt så kolossalt. Vi försökt att äga något vi endast haft till lån.	Stänger in mina känslor och blir lite handlingsförlama d. Men stundtals hoppfull. Hoppas att detta ska kunna bli en väckarklocka för människor och stärka solidaritet och empati.	Försöker gå ut så lite som möjligt trots att jag är frisk. Tvättar mig ofta Och håller distans till andra. Träffar ej äldre familjemedlemmar		Är mer lugn för min egen och familjen skull jämfört med början av pandemin just nu eftersom vi vet att det inte drabbar vår kategori lika hårt.<br>Just nu känner jag större oro för världsutvecklingen och hur den går i en neråtgående spiral och göms i pandemins spår.<br>Fler högerextrema åsikter normaliseras, misogyna förtecken, hot och hat i hemmet etc. Jag är orolig för hur detta kommer att påverka mitt barns framtid.<br>Kommer det att komma fler hot som detta i framtiden. Har vi lärt oss något egentligen?<br>Tvätta händer, sprita och att allt är mycket mer omständligt är normalitet och jag har liksom nästan glömt hur det var innan pandemin.<br>Men längtar oerhört till att kunna krama och ta på allt och alla.<br>Hoppas kunna göra mer av detta i framtiden :-)	Kvinna	1981

18<br>7	2020032<br>1	Jag är mest rädd över att bli allvarligt sjuk då jag har tvillingar på ett år som behöver mig och är orolig över att dö, har fått dödsångest, och orolig över att förlora de som står mig nära.	Jag får panikångest attacker flera gånger om dagen och det gör att jag inte kan vara mig själv	Ja, jag skakar när jag ska till jobbet, är rädd för att vara nära andra människor och rädd för att lämna hemmet. Mitt liv är begränsad.	Jag är fortfarande rädd för att förlora mina nära och kära och att själv drabbas hårt av viruset då det verkar som att den slår väldigt olika. Det jag fruktar mest är att enbart vara en siffra i statistiken då den siffran inte bra är en siffra utan ett liv. Oavsett underliggande sjukdomar eller inte så hoppas vi alla på att få leva livet och vara frisk så länge det går. Mina barn är snart två år och jag vill se de växa upp! Det är jag mest rädd över wtt få missa!		Kvinna	1988
19<br>6	2020032<br>2	Den ekonomiska kris som jag tror kommer efter Corona viruset. Alla lån ska betalas av oss alla på något sätt.	Det känns ok, håller mig sysselsatt o följer alla rekommendation er	Är inte rädd för att bli smittad eftersom jag är en frisk 70 åring. Blir jag smittad så tror jag att jag kommer att klara mig. Jag håller mig isolerad som jag ska enligt myndigheterna	Nu är jag mest rädd för att jag aldrig eller att det dröjer länge innan jag får åka och hälsa på min dotter och hennes familj som bor i USA. Det är lite sorgligt, till jul blir det ett år sedan vi sågs. Vi brukar ses två till tre gånger om året. Men annars, ja, jag följer alla rekommendationerna och blir jag sjuk så blir jag. Är faktiskt riktigt trött på det här och besviken på alla de som bryter mot våra restriktioner. Så känner jag det, nu ska jag ut på promenad i det vackra höstvädret.		Kvinna	1949

197	20200322	Jag är rädd för vad som kommer att hända dem som bor på platser utan vård (eller ens vatten), som i flyktingläger. Det här kommer att drabba fattiga riktigt hårt. Jag ser att det finns potential att vi i den rika delen av världen skiftar perspektiv, blir tacksamma för vad vi har, uppskattar välfärden, samarbetar, är solidariska med varandra och betalar mer skatt. Samtidigt finns en rädsla för att mer främlingsfientlighet och vi-och-dom-tänk kommer med den ekonomiska krisen. Jag hoppas att samhället ställer om till ett bättre, att det inte går tillbaka till det vanliga, men jag vill inte att alla som redan går på knäna ska behöva lida på vägen dit.	Jag läser nyheter flera gånger om dagen, vilket jag inte brukar. Jag hör av mig till vänner och familj, även släktingar jag sällan pratar med. Jag är lite på spänn.	Mest av rädsla för att smitta andra i riskgrupper, ifall att jag skulle vara smittbärare utan att vara sjuk. Jag gick till andra sidan cykelvägen när en gammal dam kom med rullator och munskydd, men hon trodde att jag var rädd för henne, stackarn. Hon försökte förklara att hin inte var sjuk. Jag köper mest mat online, försöker undvika affärer, bibliotek och folksamlingar, studerar hemma. Läser alla rekommendationer från myndigheter. Blev tidigt insatt i Corona för att jag var en av de första som sattes i karantän i Skåne, innan det blev pandemi, för att jag hade varit på en fest med en smittad student. Ingen av oss på festen blev sjuka, men fick sitta 14 dagar i karantän. Vi fick gå ut i naturen, så jag och min dotter var ute i fem olika naturreservat. Så mycket brukar jag aldrig hinna se av våren såhär års!		Beklagar att jag inte kan besvara detta för jag måste begränsa datoranvändning pga hjärnskakning.	Kvinna	1985

200	20200322	Att min man och dotter som är fast på Irland respektive Turkiet blir sjuka och jag inte kan åka och hjälpa dem.	När jag blir rädd försöker jag tänka på rädslan i bitar. Då går den oftast sönder och jag är inte rädd mer.<br>Jag tänker också på fina saker jag gjort med min man och dotter.<br>Jag gör saker för att minska dåliga tankar. Städa är bra. Handarbeta också bra. Lyssna på radio bra!	Nej	Inte så stor förändring inte rädd!<br>Lite mer trött på situationen och fortfarande trött på människor som blir galet oroliga och därmed okonstruktiva, flera av dem håller sig dock hemma (arbetskamrater) men tänker att många kan börja må riktigt dåligt. Känns inte bra är eg nog mest rädd för dessa människor. Känns som de potentiellt har stor förmåga att påverka samhället negativt på många plan.<br>Det känns lite extra tråkigt när det nu känns tydligt att detta kommer pågå lång tid framöver. Vad gör jag med mamma och pappa 80+? Försöker hitta på saker men nu när det börjar bli kallt är det mindre lätt. Får bli soppa och glögg ute samt promenader, vilket ju är bra för alla!<br>Familjen inga större förändingar barnens skola och aktiviteter fortsätter. Har min man mer hemma vilket är kul!<br><br>Också tydligt att det är svårt att ändra arbetsformer så vi kan få det positiva med social interaktion. Vissa gillar inte zoom (zoomar ut) andra är kass på att ordna bra zoom möten (som känns		Kvinna	1968

					trevliga) Men kanske är det en läroprocess känns som det finns små tecken. (tex vissa styrelsemöten eller liknande som alltid är formella, kan med behållning hållas på zoom och bli kortare möten för det mesta. Det är bra.<br><br>Men känns som vi potentiellt står inför en möjligt positivt förändring av hur vi arbetar (i alla fall vi som har skrivbordsjobb) men krävs att chefer kan utveckla vad som är bra och minska det mindre bra samt vara lyhörda för att vi är olika och behöver olika saker.<br>Så rädd, nej kanske för de rädda. Mina föräldrarss levnadskvalitet har förbättras på flera sätt (mer aktiva men visst ite trevligt om de blir sjuka) men varken jag eller de är rädda. Vi gör det bästa av situationen.			

202	20200322	Att jag ska bli smittad, eftersom jag tillhör en riskgrupp	Jag blir rädd, vilket är en ovanlig känsla för mig, samt irriterad över inskränkningarna jag måste göra	Ja, jobbar hemma, undviker att träffa folk med yngre barn	Jag är rädd för att någon i min närmsta familj ska drabbas. Jag och maken har båda problem med lungorna, att få covid-19 skulle troligtvis vara dödligt för oss. Samtidigt har vi barn i skolåldern, högstadium och gymnasium, så risken finns att de bär hem viruset, trots att vi föräldrar isolerar oss.		Kvinna	1966

204	20200322	Att förlora någon i familjen. Att själv bli svårt sjuk och mina barn kanske förlorar mig.	Ångest och ångestattacker.	Är försiktigare...Mina barn och jag har hållt oss inne och det tar hårt mentalt.	Kände mig länge väldigt trygg med att bo i vårt Skåne. De människor som väl vistades ute visa respekt och höll avstånd.<br>Man blev nästan på chockad över hur tomt det var på gator och i affärer.<br>Jag undvek att handla med mina 4 barn i 6 månader och de få gånger vi var iväg reta jag mig bli på alla dessa personer som inte förstår meningen med munskydd och handskar.<br>Folk fingra på sina munskydd, drog dom under näsan. Pilla på saker klia sig i näsan m.m. sen på med skyddet. De med handskar och munskydd som höll i sin mobil med handskar och som dom röka en cigg med handskar på och munskyddet under hakan.<br>Dessa önska jag att dom förstod hur man använder dessa skydd.<br>Dock kände jag att jag inte kan styra över den biten att det är deras misstag.<br>Mina barn och jag har inte bjudit hem någon sen Covid-19 spreds i Sverige och vi höll och friska tills (peppar peppar) tills skola/dagis börja.<br>Sen augusti har vi haft 3 förkylningar.<br>När Covid-19 på nytt stegra i Sverige kände jag återigen		Kvinna	1976	

					att det är tur vi bor i Skåne. Plötsligt ser man hur folk börjar strunta i att respektera Folkhälsomyndighetens rekommendationer. Folk går i butiker fastän dom är genomförkylda, dom står ändå in på en och blir irriterade om man ber dom hålla sig 1,5 m bak.<br>Där kom min rädsla på nytt!!<br><br>Jag är livrädd för att barnen eller jag ska få Covid-19. Vi har alla nedsatt immunförsvar, alla har astma.<br>Vi undviker shoppingcenter, jag handlar i mataffär ensam.<br>Min magkänsla brukar inte ha fel...<br><br>Jag har beställt munskydd till mig, till barnen och min mamma. Hädanefter kommer vi använda detta om vi är i folkmassor.<br><br>Jag har sen 2013 fått enorma problem med mina lungor. Får ofta Astma anfall och lunginflammation.<br>Är rädd för att inte överleva om jag blir smittad.<br><br>Jag läser på Region Skånes hemsida om ökningen i Skåne och känner hur det			

					kryper i skinnet på mig.<br><br>Läste att skolorna har ingen skyldighet att informera om det finns Covid-19 hos personal eller elever (som förhoppningsvis är hemma sjukanmälda). Och på den biten blir jag arg. Det är en pandemi vi har, dom borde vara skolans plikt att lämna ut information till oss föräldrar.<br>Läste i tidningen om att smittan finns på flera grundskolor här nere och jag blir både rädd och arg.<br><br>Jag hoppas HOPPAS att vi snart får leva som vanligt utan Covid-19 flåsandes i nacken.<br><br>Jag är nu rädd för att leva och det känns skit. Covid-19 har format allt vi gör.<br>Blir det värre, vilket jag tyvärr tror kommer jag frivilligt isolera min familj igen.<br>Har sagt till barnen... får jag höra av någon förälder att Covid-19 finns på skolan så släpper jag inte iväg mina barn. Skolan kan hota mig hur mycket dom vill med att anmäla mig till soc.<br>Mina barns hälsa går före allt!!!			

208	20200322	Att jobbet inte vidtar åtgärder för att skydda sina medarbetare. Sjukvården. Att jobbet är för sent på bollen. Som en för lång kedja med senfärdiga myndigheter, regioner, sjukhusledning och golvet - tar för lång tid att implementera vettiga beslut. Internationella nyheter vittnar om var vi kommer att befinna oss om några veckor. Men ledningen beter sig som om allt är som vanligt.	Inget ovanligt eller unikt.	Frivillig isolering. Förkyld.	Coviden försvårar möjligheterna att söka andra jobb.		Kvinna	1962
210	20200322	Jag är rädd för att människor jag älskar kanske kommer att dö. Eller att jag ska dö. Är inte i känd riskgrupp personligen men det är ju omöjligt att veta hur sjuk man blir. Är rädd att sjukvården ska kollapsa helt. Är rädd att vi ska förlora våra jobb och inte kunna klara oss ekonomiskt.	Jag får lätt ångest, har svårt att tänka logiskt och får svårt att andas lugnt. Gråter ibland när barnen somnat. Försöker låta bli att tänka för mycket.	Jag jobbar i butik och ser många hosta/nysa i handen fortfarande. Jag använder handskar så mycket jag kan, försöker hålla ett större avstånd än vanligt. Tvättar mig ofta och länge. Har börjat vända huvudet lite åt sidan när jag pratar med någon så att saliv inte ska råka flyga i ansiktet. Hostar och nyser i armvecket om jag behöver. Undviker att umgås med folk just nu. Hoppas på en fin vår så man kan ses utomhus.	Jag är rädd för att bli svårt sjuk och/eller dö ifrån mina barn. Eller att någon som står mig nära ska bli svårt sjuk eller dö. Det är också tråkigt att människor bråkar och inte "tycker lika", dvs många som inte lyssnar på myndigheternas råd. Är orolig för att folk inte tar detta på allvar.		Kvinna	1975

222	20200322	Att samhället stannar still och inte kommer igång igen. Jag är också rädd för min dotter. Hon föddes utan immunförsvar i sina slemhinnor. Att även om jag haft alla symptom på covid-19, då får jag inte testa mig, för som vården sa: har du inte skakat hand med hen, då har du inte smittas. Nej, men jag jobbar tillsammans med hen och har delat på handtag, pappar mm och hen kom hem från skidsemester i Österrike och blev sjuk 1 vecka efter hen kom hem.	Vågar inte att bjuda min dotters pappa hem till middag. Han är svårt överviktig och har diabetes. Jag försöker inte att gripas av panik.	Jag har blivit tvungen att jobba hemifrån. Min arbetsgivare vill inte att jag ska ta bus och tåg till Köpenhamn där min arbetsplats finns. När jag inte fick testa mig, och jag blev feberfri har jag försökt återgå till återigen att handla normalt. Ingen hamstring.	Hejsan, min första tanke var att jag inte hade något att våra rädd för, men jag hade inte tid att skriva just då. Under kvällen smög sig en tanke in i huvudet på mig. Tänk om det skulle bli - här i underbara Skåne - som i Danmark. En strategi som tar sitt ursprung i hur populär skulle jag som politiker bli, om jag röster fram krav på ditt och datt. En strategi som inte grundas i forskning och evidens.<br>Jag är inte alltid överens med Anders Tegnell, men hans strategi är best för samhället.<br>Så jag är faktisk rädd att riksdagen röster fram en ny pandemi lag, som gir regeringen makten att bestämma om vilka begränsningar som ska vara. Jag tycker jag märker, att politikerna gärna vill som kollegerna i resten av Europa, nämligen bestämma över skickliga ämbetsmän och forskning.<br>Jag jobbat i Köpenhamn och är nu igen på ”köks kontoret” och kan se fram till igen att få komme på kontoret nån gång till sommar (2021) och om jag ska stämma träff med mina syskon (som bor i DK), skal vi välja mellan vilka av våra barn som får närvara. Vi får		Kvinna	1961

					vara maximalt 10 personer i privata hem.<br>Jag fattar helt enkelt inte att en regering får besluta att belasta miljön med så mycket engångsplast som bara slängs i naturen. När jag tar min dagliga promenad i mitt bostadsområde, har jag redan sett att munskydd finns i buskar och på gräsmattan, har även sett en mås med ett runt halsen och dens förtvivlade försök att komme los. Och det är inte ett krav att bära dem här i Sverige. De 3 veckor jag var på kontoret i Köpenhamn, så jag munskydd ligga överallt. Vilken miljökatastrof för att rädda livet på kanske 5 människor.<br>Så Ja, jag är rädd här i pandemin. Inte för att bli sjuk, men för maktbegäret och dens påverkan på samhället och på hela världen.<br>Den världen som våra barn ska leva i. Hur får dem inte tänka om 30 år? Jo man räddade jo livet på 5-10 människor och tänkte inte på framtiden. Nu får vi leva med restriktioner på mat, för att där helt enkelt inte finns nog. Fiskar svälter ihjäl, för att de äter plast. Grönsaker kan inte odlas i			

					plasten i värme länder. Tur man bor i Norden med tillgång till odlingsmetoder som faktisk gir möjlighet att vi kan bli mätta. Hur tänkte dom egentligen den gången i 2020 ??			

226	20200323	Efterföljderna av den så kallade Coronakrisen. Min förhoppning är att människor inser hur viktigt det är att vi hjälps åt och delar med oss; tänker på varandra. Men det kan också bli tvärtom. I svåra ekonomiska depressionstider kan diktaturer uppstå.	Blir stel, både rörelsemässigt sätt i kroppen samt i tankara.	Nej. Men jag tar det säkra före det osäkra och håller avstånd för att inte smitta någon annan ifall jag bär på viruset utan att känna av det.		Mitt svar på frågan vad jag är mest rädd för just nu, är att jag oroas över hur pandemin kommer drabba samhället och världspolitiken i stort. Alla länders ekonomier påverkas. Kommer vi få uppleva en ny fattigdomsepok i Sverige? De generationer som lever idag har inte varit med om de svåraste tiderna som svenskar upplevde under statarperioden, när drängar och pigor (inklusive barn) slet på gårdar och gods, i smedjor, gruvor och glasbruk. På byggen...med mera.<br>Vi står samtidigt inför en klimatkris som slår globalt. Orkaner och översvämningar blir allt vanligare.<br>Vi har stormakter såsom Kina och Ryssland och diktaturer såsom Belarus som hotar och förtrycker sina befolkningar. Kommer några stormakter, tex Kina passa på att ta världsherravälde när länder försvagas av hanteringen av pandemien?<br>Med Coronan som spridits över jordklotet underlättas knappast människors frihets- och jämlikhetskamp.<br>Kommer människor orka med påfrestningarna? Kommer vårdpersonalen på sjukhus, vårdhem och inom hemtjänsten orka jobba vidare? Några av dem har blivit utförsäkrade av Försäkringskassan efter att ha insjuknat i svår Covid 19.<br>Hur kommer människor bete sig när arbetslösheten ökar, när fler kanske måste flytta från hus och hem? När tvångsremmarna dras åt till bristningsgränsen och vi eventuellt också börjar lida brist på mat, ifall	Kvinna	1959

						hushållsekonomin inte räcker.<br>Kommer människor klara av att samarbeta så vi kan hjälpa och bistå varandra i stället för att slå ut varandra i ett scenario, liknande Rysslands efter Sovjetunionens fall, där blivande oligarker köpte upp konkursdrabbade företag och blev miljardärer och förmögna på andra människors bekostnad i ett korrupt och kriminellt styre?<br>Kommer barn och unga här i Sverige, klara av att ta igen missad skolgång? (Alla har inte fixat att studera på distans.)<br>Kommer vi bli mer begränsade i våra levnadssätt? Mer bevakade och kontrollerade? Kommer isolering, ensamhet och sorg bre ut sig och knäcka folk psykiskt?<br>Kommer polariseringar och ve och fasa - eventuell fientlighet - människor emellan öka? Utbredd rädsla hos befolkningar har aldrig varit gott.<br>Jag ser redan negativa effekter jag inte hade räknat med skulle uppstå när pandemien var "bara barnet.", i mars i år. Människor börjar må dåligt.<br>Jag hyser en förhoppning om att vi människor är kloka nog att närma oss varandra mer i stället för tvärtom och inse att vi behöver varandra. Må det vara naivt - men något måste man tro på. :)			

228	20200323	Att man inte håller sig till direktiven och stannar hemma. Man har inte tålamod att frivilligt isolera sig, utan man måste ut i folksamlingar, gå på puben m.m	Kan få ångest!	Ja, frivillig isolering. Håller avstånd. Följer direktiven som ges.	Inte rädd för min del. Rädd för att man inte respekterar rekommendationerna. Det som inte förstår att vi är mitt i en pandemi,dem är jag rädd för.		Kvinna	1958
230	20200323	Att få Corona då jag har kronisk astmabronkitis sedan jag var barn. Normale människor kan pusta 8 liter, jag kan pusta 2,3 liter. Jeg har två pojkar på 8 och 10 år.	Jag blir frustrerad och arg på att så människor kring mig inte tar detta på alvor.	Inte mycket då vi bor på landet och inte har så många människor kring oss. Från i dag 23 mars går jag och alla mina kollegor dock från full tid jobb till 40%, då vi förlorar pengar. Hurra för den svenske regering som tog beslutet i torsdag om att hjälpa verksamheter som har ekonomiska utmaningar under denna kris.	Det er intet något jag som sån går och tänker på dagligen. Jag gör det som där rekommenderas att vi ska göra. Dock om jag tänker på det, kan jag bli rädd att någon smittar mig för att de inte passar på, då jag har astma bronkit och därför inte tåler att bliva sjuk. Tyvärr har jag unga kollegar som inte tar det med corona på alvor och därför inte bryr sig. De VILL dansa och dricka på stan bland alla andra, och det gör mig ibland arg och ledsen, dock inget som fyller dagen.		Kvinna	1971

23 1	2020032 3	Jag är rädd att jag eller min man ska bli allvarligt sjuka och inte kunna ta hand om våra barn. Vi behövs båda två i omvårdnaden då vårt ena barn är 10 v och vår 4 åring har autism.	Jag får dåligt tålamod med barnen, jag sover dåligt o drömmer mardrömmar, minskad aptit.	Ja, vistas mindre utanför hemmet i närområdet. Läser mer nyheter. Träffar vänner och familj sällan.		Är fortfarande rädd att jag eller min man blir sjuka, att inte kunna ta hand om våra barn. Är rädd för man tar fler risker nu än i början av pandemin för man har blivit "isolering trött". Var livrädd med ångest i våras men det är jag inte längre, hade jag varit så rädd fortfarande hade rädslan gjort mig sjuk.<br>Jag är just nu också rädd för att inte kunna skaffa arbete efter min föräldraledighet pga situationen.	Kvinna	1985

23<br>8	2020032<br>3	Att folk inte förstår budskapet och gör som alltid.	Jag oroar mig sällan an. 60%av det man oroar sig för kommer aldrig att hända. Och av de kvarvarande är det bara 7%enheter som vi själva kan påverka.	Jag använder handskar, nya för varje patient, även om man inte anser dem smittade.	Jag är inte rädd. Det finns en början och ett slut på livet. Vi kan inte påverka det första och knappt det senare heller.<br>Det som förvånar mig är att folk har kopplat bort det sunda förnuftet och insikten i historien.<br>Pandemier stora som små påverkas inte av facebook och filterbubblor utan av en logisk utveckling. Titta på historien, Spanska sjukan, HIV / AIDS, Hongkong, Asiaten, Svininfluensan, MERS, SARS mfl de försvinner inte bara, de finns kvar i samhället och återkommer under flera år.<br>Jag är inte förvånad av den andra vågen nu i oktober 2020. Jag tror att det kommer en tredje våg 2021.<br>Okunskap bl a med förmodade sanningar, gör att vi tror en massa, men vet så lite. Varje gång som vi tror att vi kan behärska naturen, bedrar vi oss själva. Varje gång som vi tror att vi på konstgjord väg kan bemästra naturen så ökar vi spridningen av virus.<br>Jag är rädd för människans dumhet eller godtrogenhet.<br>Jag vill att vi skall vara försiktiga och rädda om varandra, inte rädda för varandra.		Man	1958

243	20200324	För att jag som är i riskgrupp och min son ska bli sjuka i Covid-19	Jag har ångest och lider av stress så den är lavinartad. Mina muskler rycker och skakar, jag får sjukdoms symptom och blir utmattad minst sagt	Vi är isolerade och vi tvättar händer och allt frenetiskt.	Vad jag är mest rädd för? Att jag eller någon närstående ska få covid 19 och dö eller få bestående men. Det är en daglig oro som orsakar stressrelaterade problem för mig och iom pandemin så får jag ingen hjälp inom psykiatrin trots remisser och EXTREMT behov. Min kropp lägger snart av känns det som och jag står här och skriker efter någon som kan hjälpa mig men är lämnad åt min ensamhet. Det är fruktansvärt. Jag vägrar ge upp men varje dag är en hemsk kamp mot mig själv och mina tankar.		Kvinna	1983

246	20200324	Att inte klara av smittan och att det inte finns nån beredskap i Sverige att hantera nån kris överhuvudtaget. Myndigheterna ljuger och kommer med motstridiga besked. Har ringt 1177 och jag måste vara halvdöd innan jag provtas. Att ha alla symptom räcker inte. Det känns otroligt otrygg!	Jag söker så mycket information så möjligt från utländsk media. Varken svensk media eller myndigheter inger en känsla av trygghet. Staten och myndigheterna tänker mer på ekonomin än medborgarnas hälsa.	Ja! Jag åker varken buss eller tåg. Äter inte på restaurang samt undviker folksamlingar. Har handlat mat så det räcker för 2 veckor i taget. Är noggrannare med handhygien. Följer mer än noggrant föreskrifter på jobbet för att undvika att smittas.		Jag är mest rädd att Folkhälsomyndigheten FHM tänker mer på ekonomin än på vår hälsa. Strategi mot munskydd är för mig obegripligt! Igår 19/11 fick jag veta att FHM anser att munskydd är nära på en klass fråga och att de tycker att köpstarka grupper kommer att använda munskydd mot att andra mindre bemedlade grupper inte kommer att göra det. I andra länder är det staten som står för kostnaden! Jag är rädd för att staten och myndigheter är senfärdiga med åtgärder som kommer att skydda allas liv på lika villkor. Sverige trots att vi är mitt i andra vågen har inte lärt nåt från andra länder! Tester kan göras och på 15 minuter få svar om personen är smittad men här måste man vänta flera dagar! Jag är också rädd eftersom FHM har tillåtit allmänspridning i den så kallad flockimmunitet! nu måste jag opereras och kanske får jag inte vård pga alla resurser går till corona patienter! Nu är det för sent med kraftfulla åtgärder och Sverige har visat sig vara det land som tillsammans med USA och Brasilien mer har struntat och nonchalerat pandemin, och ljugit hela tiden! Dödstalen stämmer inte. Den 19/11-2020 fanns 19 personer som avlidit av corona men bara i Skåne fanns 23 personer avlidna.	Kvinna	1956

247	20200324	Jag är rädd för att samhället ska kollapsa, att min kommunala jobb inte ska kunna betala ut min lön om några månader. Att vårt hus inte ska vara värt någonting om några månader och att vi därför inte har någonting.	Jag får ont i magen samtidigt som jag bara kan lägga rädslan åt sidan och tänka att det inte är så nu och att vi får ta det när det händer. Om det händer.	Ja, tvättar händerna mer och slutat ta i hand. Håller större avstånd på jobbet (jobbar på en högstadieskola), men det handlar nu om 50cm istf 30, så ff ganska nära andra människor hela tiden. Massor med aktiviteter i ställda, så mer tid med familjen. Vilket är skönt och roligt.	Hej! Förra gången minns jag att jag svarade att jag var rädd för att samhället skulle kollapsa och att vi skulle bli av med huset. Nu handlar min rädsla mer om att någon i min familj ska bli sjuk och att skulle vara mitt fel. Jag tror hela tiden att jag har symptom och att jag ska smitta andra. Det är som att jag tror att jag när hela världen på mina axlar.		Kvinna	1976
260	20200325	Att dottern, som tillhör riskgruppen, ska bli sjuk. Att 70+arna inte ska ta sitt fulla ansvar.	Lite ångest.	Är 70+are. Alltså frivillig karantän.	Jag är nog mest rädd för att 70+arna inte inser allvaret i och med att restriktionerna ändrats och inte "håller i" så att de blir sjuka och är anledningen till att sjukvården kollapsar.		Kvinna	1946

26<br>1	2020032<br>5	Att mina föräldrar skall bli sjuka de är båda äldre , sjukliga och ensamma	Får dåligt samvete och stresssymtom och orkar inte ta kontakt	Undviker att vara ute bland folk efter jobb		Det jag är mest rädd för nu är att:<br>mina föräldrar skall smittas och att jag inte skall orka hålla ihop mig själv då ingen hänsyn tas till skolpersonals situation och arbetsbelastning . Vi skall agera poliser, städare, sjukvårdspersonal, extra vikarie, samtidigt som vi skall ha undervisning för klasser som inte är fulltaliga , se till så att alla kan jobba i kapp, hjälpa och anpassa all undervisning , motivera konstiga beslut och sköta sin egna säkerhet så gott det går. Det sätter sig på humöret och jag blir folkskygg	Kvinna	1961

262	20200325	Är generellt inte särskilt rädd. Är ung och frisk och tillhör inte en riskgrupp. Det man är rädd för är att man skulle bära på viruset och råka smitta någon annan. Särskilt min mormor eller farmor och haft det på sitt samvete...	Man blir försiktig och försöker följa de råd som kommer.	Man är mer hemma då skolan har stängt och man tänker lite på att vara på platser där det kan vara mycket folk. Men dock har man fått en liten väckarklocka om livet man lever. Det mesta som man har haft planerat har varit nöjen som man nu märker att man kan leva utan, även om det blir lite tråkigt, men också att man får mer tid att ta hand om sig själv. Har fått tid att läsa mycket böcker och rört mig mycket ute i naturen där tankarna har gått åt en själv.	Det jag är mest rädd för just nu är att man inte ser något slut på pandemin. Vissa restriktioner har lättat men fortfarande är smittspridningen stor. Det har varit påfrestande att vara mycket på distans och man är rädd för hur man ska hantera sin egen psykiska hälsa med den större ensamheten, särskilt nu när man dessutom är inne i de mörkare månaderna.<br>Jag är även rädd om mina mor- och farföräldrar. Dels över att de ska bli sjuka men också vad isoleringen gör mot dem. De lever inget roligt liv och känns jobbigt att det ska vara så när de är i sina sista år av livet antagligen.<br>Jag har även en rädsla för vilka splittringar som sker i samhället och all debatt som uppstår mellan många icke-experter kommer göra att pandemin pågår längre. Tillexempel den återkommande debatten om "hur kan man vara flera tusen på varuhus samtidigt som kultur och idrott inte får ha publik". Jag är rädd att dessa, ibland ologiska, restriktioner gör att folk kommer tröttna och inte ta restriktioner på lika stort allvar. Vilket man redan nu ser.		Kvinna	1996

26<br>5	2020032<br>5	Att trenden med människor runt om i världen, och ledsamt nog även i Sverige, som tycks längta efter snabba fascistoida lösningar på komplexa problem ska kunna få ett uppsving i och med aktuell pandemi.	Blir beklämd och ledsen. Får tankar om att vi som art inte förmår bättre. Människor är en social art, precis som myror och bin. Jag kan få tankar om att vi som art är biologiskt oförmögna att reglera våra samhällen på andra sätt än destruktiva, vilket historien så ofta verkar visa oss... Samtidigt kan också en känsla av ilska och kamp väckas!	Jag är väldigt noga med att följa FoHMs rekommendationer. Dels för att skydda våra svaga grupper och sjukvården, men också av en stark vilja att visa att tillräckligt många av oss har förmåga att visa ansvar och solidaritet. En annan stark känsla just i nuläget är djup tacksamhet - och stolthet - över att vi har så oerhört kunniga experter inom våra svenska myndigheter men också politiker som har modet att följa deras råd i stället för att visa prov på missriktad handlingskraft.		Jag är nog fortsatt rädd för likartade saker som i våras. Starkare motsättningar i samhället och antidemokratiska/fascistiska/faktaresis tenta grupper som spinner loss på otrevliga vis. Att även etablerade nyhetskanaler lutar alltmer åt sensationssökande clickbaitjournalistik. Vill minnas att detta var sådant som skrämde mig redan i våras.<br>Sedan har en mer personlig rädsla infunnit sig den allra senaste veckan också. Jag har en son som driver en barkedja. Vilket har varit ett hårt och svårt arbete det senaste halvåret. Han och resten av ledningen, varav många står mig nära, har kämpat oerhört för att kunna bedriva sin verksamhet i fem städer på ett smittskyddssäkert sätt. Nu när ljusningen ledsamt nog tycks mer avlägsen än vad vi väl alla hade hoppats på och regeringen tar till krafttag mot just krognäringen känner jag en rädsla och oro för hur det ska gå för honom och hans företag...	Kvinna	1964

266	20200325	Jag är rädd för att gå i personlig konkurs och för att sjukvården ska bli så överbelastad att alla inte kan gå hjälp. Sen är jag rädd för människors egoistiska och avståndstagande sida som krisen framkallar.	Jag tänker inte så mycket aktivt på det, men det ligger som en gnagande oro. Jag drömmer mycket mardrömmar om hur jorden går under.	Jag tvättar mest händerna väldigt noga.	Jag är fortfarande rädd för att jag ska råka smitta andra, som kanske smittar någon i riskgrupp. Jag är också orolig för att världen inte kommer bli densamma igen. Att "Efter corona" inte finns på det sättet vi tror.		Kvinna	1996
267	20200325	Att bli svårt sjuk av corono. Det gäller hela min kärnfamilj. Jag är rädd och orolig.	Jag blir stressad, försiktigt, håller oss isolerade.	Isolerar familjen. Känner mig smutsig efter att ha varit i affären 1 gång/ veckan. Planerar inköp noga. Håller avståndet.		Mest rädd för att familjen ska bli smittad av covid och att någon av oss blir allvarligt sjuk.	Kvinna	1973

268	20200325	Ekonomisk kollaps. Talen för viruset redovisas inte rättvist. Hänsyn till mörkertalet har inte tagits i samband med redovisning av dödsprocent i media. Detta har skapat panik hos många som inte är kapabla till dataanalys. Självklart ska vi ha respekt för viruset, trots låg dödsandel (0,5 till 1%, om ens det), eftersom många blir sjuka samtidigt. Det är en överbelastad vård som är farligt inte viruset i sig. Total avsaknad av efterfrågan i många branscher är att ta i... Sydkorea är ett stort föredöme som satsat på masstestning - Skapar trygghet eftersom man fått koll på om man är smittbärare eller ej. 50% av smittbärare i genomförda masstester har ej uppvisat symptom eller haft mycket lindriga...	Klump i magen. Letar fakta.	Nej. Jag är inte rädd för att bli smittad av viruset. Följer bara myndigheternas rekommendationer och har stannat hemma vid minsta förkylningssymptom. Varit ute på promenad med hunden dock och handlat mat. En sjuk måste också äta...		Människor som fattar beslut grundat på rädsla istället för logik. Samhället lider av masspsykos just nu. Väldigt otrevligt. Mycket få människor som har förmåga att tänka klart känns det som.	Kvinna	1989
272	20200325	Inget speciellt - tar dagen som den kommer.	Inget - blir inte rädd	Följer myndigheternas rekommendationer och behåller sunt förnuft	Jag är mest rädd för att någon anhörig smittas.		Man	1956

278	20200326	Den pandemiska paniken...	Det handlar om informationsflödet. För lite information sätter igång fantasin - oftast till det värre. För mycket information späder på paniken. Rädsla innebär ofta att man stänger av och/eller agerar i panik. Personligen försöker jag stänga av eftersom jag befarar en överreaktion i världen där vi slår undan benen på oss själva innan viruset gör det...	Jag är medveten om att hålla avstånd, spritar händerna, nyser i armvecket (när jag kommer ihåg) men i övrigt lever jag som vanligt. Jag försöker följa med i nyhetsutvecklingen så gott det går och klura på varför likartade länder agerar på så olika sätt. När det här är över har vi nog lärt oss en del nytt om pandemier och hur vi framgent ska förhålla oss utifrån ett globalt perspektiv.		Jag är mest rädd för att bli sjuk "i onödan" precis före vaccinet blivit klart. Då jag tillhör riskgruppen kommer jag med största sannolikhet att vaccinera mig.	Man	1966
285	20200409	Att inte kunna få anställning och försörja mig. Var mellan jobb innan krisen. Är högutbildad men känner av diskriminering samt stopp just nu i anställningsprocesser som är minst 4-5 mån långa med tester, Interjuer och säkerhetsprövningar och nu tröghet med virus hysterin. Att inte få tillbaka ekonomisk trygghet är värre än att få viruset.	Har varit deprimerad pga andra arbetsrelaterade psykosociala orsaker. Orkar inte vara rädd. Rädslan har övergått i en känsla av hopplöshet. Orkar inte	Jag följer de allmänna råden men isolerar mig inte. Det har jag gjort tidigare i ett deprimerande tillstånd. Det räcker nu att vara rädd. Att vara konstruktiv är bättre.	Det jag mest känner oro är att inom landet och globalt kommer att öka spänningar i samhällena pga ekonomisk stagnation som en konsekvens av nedstängning av samhällen och länder. Krisen blottar samhällets svagheter och sårbarhet. Också ser jag risk för en återgång i utvecklingen för ekonomisk tillväxt, jämställdhet m.m.		Kvinna	1969

288	20200409	Att mina närmaste ska bli allvarligt sjuka. På grund av ett tidigare för tidigt dödsfall i familjen (före corona) så har jag allmänt blivit mer hälsofokuserad och lyssnar på min kropp. Med viruset så aktualiseras detta igen. När man själv och ens närmaste är friska så glömmer man döden på nåt sätt. känslan när man var obekymrad om döden kommer jag nog inte uppleva igen. I och med det tidigare dödsfallet så är jag mer medveten.	Jag tänker mycket, har ibland svårt att sova.	Jag isolerar mig så mycket jag kan, gör hela tiden sannolikhetsövervägan den när jag går ut (inga trånga utrymmen, inga folkmassor, avstånd). Nöjen som bio och konserter är inte tillgängliga så att vara ute i naturen på egen hand eller med familjen har blivit vanligare. Jobbar hemma som vanligt (företagare), så inga förändringar där och jag har mycket jobb i och med att jag jobbar i läkemedelsbranschen.	Att min familj ska bli svårt sjuk och att att det sociala livet ska begränsas permanent. Eftersom vi hittills klarat oss utan corona inom familjen så är jag väl nästan mer rädd för att kulturlivet som konserter och teatrar ska begränsas permanent. Jag känner det som frustrerande och en källa till att tycka hela livet känns tråkigare nu. Jag tror att depressionsfallen kommer att öka.		Kvinna	1971
289	20200409	Hur samhället påverkas. Att tex högerextrema krafter ges utrymme och att klimatkampen pausar/stoppas. Och att mina föräldrar ska drabbas illa av covid.	Jag är van. Corona-ångest är typ samma som klimatångest. Jag googlar för mycket och försöker hitta ljusglimtar.	Nej, men pga rädsla att smitta andra utan att veta. Dragit ner sociala kontakter tex.	Att livet aldrig ska återgå till det normala. Kommer jag någonsin få hänga i stora grupper, krama mina vänner och mina föräldrar igen?<br>Och göra saker utan dåligt samvete. Det blir ju bara värre och syns ingen ljusning direkt..<br>Och även fortfarande rädd för att mina föräldrar och svärföräldrar ska drabbas väldigt hårt.		Kvinna	1988

290	20200409	Att det blir global depression och att jobb och välfärd och det liv vi känner idag kommer att vara ett minne blott. Att många miljoner kan kastas in i fattigdom och alla framsteg man gjort för att större delen av mänskligheten har fått det så mycket bättre i välden försvinner. Tänker framförallt på Afrika och delar av Asien.	Ja mentalt känns det ju ganska jobbbigt att tänka på framtiden som inte känns som den finns med de förhoppningar man hade för bara 2 månader sedan	Jag är inte rädd för att bli smittad. Men följer myndigheter och skyddar riskgrupper. Blir jag sjuk så blir jag. Skulle jag dö så gör jag det. Det är ju inte en så stor sak för välden om jag skulle dö egentligen.	Just nu är jag mest less, jag är rädd för att man aldrig mer kommer kunna komma ut i världen på äventyr aldrig kunna resa eller aldrig kunna vara på stora evenemang som lopp (typ göteborgsvarvet etc) och konserter. Det kommer ju säkert nya virus som vi ska vara rädda för. Hur många sjukdomar har vi verkligen helt utrotat genom vacciner?, 1 sjukdom, smittkoppor... Och vi har vaccinerat mot tex polio (finns fortfarande på vissa ställen i Afghanistan) och mässling hur länge?<br>Att vi aldrig kommer återgå till en öppen och välkomnande värld igen, det är nog det jag är mest rädd eller orolig för. Det kommer bli så tråkigt, så jag hoppas verkligen att det inte blir så. och att det snart finns någon slags tidbegränsning på tristessen i pandemin.<br><br>Ekonomin, hur mycket tål det att bli lågkonjunktur i världen och vad kommer det att leda till, hur många liv kommer vi förlora globalt i svält mm pga det? Och hur många steg tillbaka kommer vi ta i fighten om fattigdomen i världen?		Kvinna	1986

294	20200409	Att bli arbetslös. Är inte rädd för virussmittan. Är mer orolig för att det inte finns tillräckligt med personal på sjukhusen om jag råkar ut för cancer eller en trafikolycka pga att de inte kan vara där för att skolorna är stängda.	Börjar tänka mer på det men försöker att låta bli då det är onödigt att oroa sig i förväg.	Ja, jag tvättar mig lite mer. Och reser mindre pga att det inte går eller är ok.	Jag är inte rädd för Corona/covid-19. Jag är mycket mer rädd för att jag eller någon i min familj ska få cancer.<br>Jag är inte rädd för att någon ska förlora jobbet för om de gör det så tror jag ändå att de kommer att få ett nytt jobb. Det är sånt som händer lite då och då. Sen tycker jag att det år väldigt tråkigt att inte få resa och se och ta del av andra kulturer. Men det är ingen rädsla kopplat till det.		Kvinna	1973
295	20200409	Andra människor. Tycker jag ser väldigt många människor som inte ens är intresserade av att lyssna på råden. I vår mataffär, som är väldigt stor, så försöker inte ens folk hålla avstånd.	Just nu blir jag mest lättirriterad.	Håller mig inne mest och försöker stor handla 1 gång i veckan. Vänder mig bort när folk kommer nära och har våtservetter med alco sprit i bilen	Jag är rädd för att personer börjar slutar bry sig. Att de inte känner att corona drabbar dem.<br>Ser dagligen personer strunta i att hålla avstånd, speciellt till personer de inte ens känner. Min mamma jobbar inom vården, de gick på knäna och var utarbetade redan innan corona men det verkar folk inte bry sig om. Såg igår en gammal kollega som åkt till Grekland för att gå på nå lär känna dig själv seminarium. Känns så onödigt just nu.		Kvinna	1988

306	20200409	Att IVA inte hinner med andra patienter. Cancersjuka, bilolyckor, hjärtinfarkter och annat.	Är lugn och mörkt pragmatisk.	Inte rädd för mig själv. Men mån om andra. Tar tex. större avstånd från äldre när ute i butiker.		Mest rädd för att få biverkningar eller andra efterskador.<br>Permanent förlust av luktsinnet, försämrad koncentrationsförmåga, sämre lugnkapacitet.<br>Ur samhällsperspektivet är orolig för arbetslöshet och isolering av alla.<br>Inte kunna komma tillbaka det normala med att skaka hand, krama, ge en klapp på axel.	Kvinna	1991

318	20200410	Att vården inte mäktar med och att patienter inte kommer att få den vården de behöver. Bla äldre inte kommer få respiratorhjälp. Samtidigt rädd att sjukvårdspersonal och personal inom äldreomsorgen inte kan skydda sig tillräckligt pga brist på skyddsutrustning.	Tankarna börjar snurra, känner mig otillräcklig. Svårt att tänka framåt och positivt.	INTE PGA RÄDSLA - UTAN FÖR ATT PLANA UT KURVAN OCH GE VÅRDEN EN CHANS ATT HINNA MED Självisolering sedan tre veckor. Arbetar hemifrån, har inte umgåtts alls med vänner (börjar däremot promenera tillsammans utomhus denna vecka), inga andra än min man och jag har varit i lägenheten på tre veckor, handlar mat vid utvalda tillfällen.		1. Nu när vi går in i en andra fas, som på många sätt ka visa sig jobbigare, är jag rädd för människornas hälsa. Arbetslöshet, oro och osäkerhet i samhället kan göra många ordentligt deprimerade. Hur ska vi klara av att hantera det?<br>2. Vårdköerna som bara växer, där planerade operationer blir framskjutna på oviss tid och för att sjukvårdspersonalen dukar under mitt i allt.<br>3. Att klimatarbetet globalt stannar av (det har det redan gjort). Vi befinner oss i ett avgörande skede i människans historia, tajmingen är djupt olycklig.4. Att jag, trots positivt provsvar i oktober efter en lindrig corona, kan bli smittad igen. Anledningen till varför jag är rädd är för att jag är precis i början av graviditet. Annars hade jag nog inte varit lika orolig för min egen del.<br>Rädslorna jag har är hanterbara, det är inte så att jag mår psykist eller fysiskt dåligt av det. Men det är en situation som är stressande för samhället i stort och det är inte roligt att veta att det kommer att pågå ett bra tag till, åtminstone ett halvår. Ja kanske tom ett år innan vi kan få någon ny form av normalläge.	Kvinna	1981

	330	20200507	Jag är orolig för de problem som kan följa på pandemin. Sociala i så väl hemmiljö som i samhället i stort, risken för krig i följden av den ekonomiska nedgången. Naturligtvis är jag också orolig för sjukdomen i sig.	När jag blir rädd agerar jag. Vid oro som det nu gäller i mitt fall söker jag efter ett förhållningssätt som verkar bäst för mig och omgivningen-	Väldigt lite sociala kontakter, de flesta via mobil eller dator. Oron är dubbelriktat, kan jag omedvetet utsätta andra för risk.	Jag minns inte vad jag svarade då men i dagsläget är de ekonomiska och sociala konsekvenserna av pandemin som oroar mig mest. Inte för min personliga del men väl för samhället och klotet som helhet.		Man	1948	

332	20200514	Att bli sjuk. Det är en sjukdom som kan vara mild som en smekning eller dödlig som ett klubbslag. Man vill inte ha den och man vill inte lämna den ifrån sig.	Malande oro och en stark förvissning att undvika alla sociala sammanhang. Kanske känns det som en social fobi skulle kunna kännas. Det är ju en form av social fobi; att inte våga, inte kunna, inte vilja möta andra.	Jag har förändrat mycket. På jobbet släpper vi inte in någon annan, än vi som arbetar här. Alla möten sker över nätet. Inga besök i butiker eller allmänna platser. Inte ens barnbarnen får komma, om de träffat ”den andra familjen” för mindre än två veckor sedan. Inga kramar. Inga systerträffar med evighetslånga möten, kantade av flera koppar kaffe, på Duvanders.	Just idag är jag mest rädd och orolig för familjens farmor som bor på ett äldreboende. Där har dom inte haft ett enda Covid-19-fall.<br>Vi har träffat henne utomhus ett par gånger under sommaren.<br>Nu skulle vi få träffa henne inne på hennes rum. Utrustade med munskydd och nyspritade händer var vi lyckliga att få återse henne och att hon kände igen oss. Mitt i pandemin och isoleringen händer det att de som har drabbats av demens förlorar minnet även av de som är närstående, förberedde oss sköterskan på.<br>Två dagar senare krossades vår känsla av att allt hade gått bra; en av oss blev rysligt matt, hade små utbrott av att vara genomsvettig blandat med frossa. Inget annat. Panik! Nu väntar vi på svar på ett Covid-19-prov och äldreboendet är informerat. Det var ett otroligt jobbigt samtal. Farmors fina kontaktperson talade lugnande med mig och förklarade att vi inte skulle ha dåligt samvete och att vi gjort allting rätt, dessutom hade ingen av oss några symtom när vi besökte		Kvinna	1963

					äldreboendet.<br>I väntan på svar svävar jag ändå mellan hopp och förtvivlan och ser inför min (fantasifulla) syn hur alla på boendet drabbas med fatala konsekvenser och vi är dom skyldiga. Hur kan man leva med det?<br>(6 timmar senare, samma dag kom följande besked: Provsvaret var negativt! Ingen Covid-19. Oändlig lättnad! Jag har ringt boendet och meddelat! Puh!)			

334	20200607	Att vi genom så få restriktioner offrar de äldre, som betalat skatt hela sitt liv. Att vi offrar dem för att pandemin inte ska förstöra för kommande generationer. Det är en fruktansvärd tanke som skrämmer mig så. Det är också oerhört obehagligt och märkligt att många inte följer de oerhört slappa riktlinjer FHM gett oss. Resten av världen sitter i karantän och här kan en inte ens förmå sig att hålla avstånd och undvika sociala aktiviteter. Det gör mig faktiskt rädd.	Jag får ont i magen, blir spänd i hela kroppen. Ont i musklerna.	Jag har minskat mitt sociala umgänge och träffar bara mina närmsta vänner och familj, framförallt utomhus. Jag har inte hälsat på vänner i andra städer. Jag har ställde in min födelsedagsfest. Jag håller avstånd till mina medmänniskor, och använder inte kollektivtrafiken. Jag tvättar händerna när jag varit utanför hemmet och undviker att ta i saker på offentliga platser. Jag försöker handla på tider då det inte är så många i affärerna. Jag är inte rädd för min egen skull att bli smittad, däremot är jag rädd för att om jag blir smittad kan smitta människor som tillhör riskgrupp.		Jag har är mest rädd för just nu är att polariseringen ökar i samhället gällande hur man förhåller sig till restriktionerna. Det känns som att det finns de som menar att man får inte göra någonting öht och sen de som bjuder in till fest, övernattning på landställe osv som om ingenting har hänt.<br>Jag tycker det är skrämmande när jag hör vårdpersonal vägra använda munskydd och säga att munskydd inte fungerar samtidigt som man i andra länder ser tvång på munskydd i miljöer där det inte går att hålla avstånd.<br>Jag är rädd för att jag måste sätta mig på kollektivtrafiken för att delta i grupparbete på universitetet, trots att det inte känns bra. Det känns ffa inte bra att bussen är full och att ingen använder munskydd, för här i Sverige funkar ju inte munskydd, vilket är märkligt. Det funkar ju i alla andra länder, vad gör svenskar så olika? Varför funkar det inte här?<br>Jag är rädd för den svenska strategin som i mina ögon är nonchalant och visar brist på ödmjukhet inför vad vi inte vet. Hur många liv ska vi offra? Det är skrämmande.	Kvinna	1992

335	20200607	Att min, i sommar 79 årige make ska bli smittad. Han tar inte så allvarligt på distanseringen. Vill jobba (i alla fall utomhus) som vanligt och besöka vissa affärer. Han tycker inte man kan leva så inskränkt.	Jag får mer migrän och annan huvudvärk. Är ständigt spänd i musklerna.	Har inte behövt ändra så mycket, då jag pga ryggproblem är mycket hemma i alla fall. Är inte rädd för att bli smittad av någon utomstående, men av min make, om han blir sjuk. Vi sover nu i skilda sovrum, då jag inte vill ha honom blåsande på mig i sömnen.	Jag känner mig inte speciellt rädd. Jag vet mycket mer om pandemin nu och vet hur jag kan undvika smitta. Jag räknar med att det blir begränsningar i den sociala tillvaron bortåt ett år till och det känns ok. Jag kommer inte ihåg, vad jag svarade i våras.		Kvinna	1945
336	20200607	Jag är egentligen inte rädd för smittan. Rädsla är ett starkt ord. Jag är solidarisk med myndigheternas restriktioner. Och vill naturligtvis även försöka undgå att bli smittad och är därför försiktig.	Jag har inte varit riktigt rädd i samband med pandemin. Men de få gånger jag blivit rädd, tex när jag tror att jag tappat nycklarna till min bostad, då blir jag stirrig och får hjärtklappning. Dock inte nu i samband med pandemin.	Jag har levt totalt avskärmad från mänsklig närkontakt, bara träffat barn och barnbarn utomhus, tagit långa promenader ensam. Aldrig träffat någon annan människa inomhus.		Två av mina vuxna barnbarn är just nu sjuka i covid 19. Jag hoppas att de blir friska snart och att de inte får kvarstående men. Själv lever jag totaldistanserad. Är 75 år och måste fortsätta så trots att livet inte är så muntert precis.<br>Jag är mest rädd om mina barn och barnbarn.	Man	1945
337	20200607	Ingenting. Det går inte att undvika en pandemi.	Blir inte rädd, bara frustrerad på samhällets oförbereddhet	Köper mera varor av oro att dom skall ta slut. Större lager hemma.		Inte rädd bara frustrerad. Kan ju inte påverka något (mer än att vara försiktig)	Man	1955

338	20200607	Min största rädsla är att antingen jag eller min make dör, eller att vi båda gör det och att barnen blir lämnade ensamma i denna oroliga värld.	Jag målar upp otäcka scenarior och kan slås av panikångest, med hjärtklappning och svårigheter att sova som följd.	Egentligen inte så mycket. Jag markerar väldigt att jag håller avstånd och förväntar mig att andra gör detsamma mot mig. Tvättar händerna mycket mer, byter handdukar oftare, trokar av handtag oftare, avstår från att träffa kompisar och släkt men f ö inget annat.	Vad jag är mest rädd för just nu är naturligtvis att bli så sjuk att jag skall behöva vård och tära på den redan ansträngda sjukvården. Att jag skall behöva vård och kanske bli sjukskriven en längre tid (har kollegor som fortfarande inte är sig själva efter att ha haft smittan i våras) och att bli så sjuk att jag inte klarar sjukdomen utan blir till en av de statistiska snart 6000 dödsfall i Sverige. Jag är också rädd att smitta andra i det fall jag blir smittad. Jag är även rädd att förlora både make och barn till denna fruktansvärda smitta. Något annat jag är rädd för att mina tonårssöner inte skall få en ungdomstid att minnas tillbaka på så som jag fick. De kommer inte få den här sorglösa tiden, inga konserter, inga fester, inga resor. Det samt att de skall komma ut i en verklighet där det inte finns jobb p g a den ekonomiska kris som kommer att drabba oss..<br>Jag är dock inte lika panikslagen som jag var för 7 månader sedan men varje dag innebär en frågeställning "om", om någon av oss skall komma hem smittade, om vi kan fira jul ihop med mormor och morfar, om vi kan planera		Kvinna	1971

					min 50-års fest nästa år, om sonen kan fira sin student och om vi alla är kvar tills dess. Det har ju blivit så mycket mer påtagligt att livet är så illa skört och kort. Mest är jag rädd för att världen och samhället kommer att bli en kall plats att befinna sig på, utan sociala sammanhang, skratt och kärlek.			

339	20200607	Att bli smittad och svårt sjuk och att någon i min familj blir detsamma. Själv har jag inte marginaler att tåla en rejäl influensa ens, så att drabbas av en svår variant av covid-19 skrämmer mig. Mina föräldrar är båda 70+ och i trippla riskgrupper, så samma med dem.	Jag blir orolig, jag äter sämre, jag är ute och promenerar extremt mycket och länge för att stilla oron. Jag begraver mig i mitt arbete och känner inte efter när jag blir trött. Jag tittar och läser för mycket på nyheter och andra faktakällor och glömmer nog bort lite att leva.	Jag lever normalt ensam, men är nog mer åt det isolerade hållet, jobbar hemifrån och är bara på arbetsplatsen en halv dag i veckan. Övrig kontakt jag har med människor är när jag måste handla mat till mig eller min hund/katt samt när jag går till min ensamarbetande frisör.	Ja vi är kvar i pandemin. Inget snabblöst problem den inte. Just nu, med skärpta 3-veckors-rekommendationer i Skåne är jag som självbo ännu mera ensam än tidigare i år (jag är dessutom i riskgrupp nämligen); jag jobbar hemma helt nu och träffar ingen "som jag vanligtvis träffar varje vecka" heller, så nu finns det stunder som är mentalt lite tuffa. Visst umgås jag med lite sällskap på en hundpromenad på helgen, chattar, mejlar och sms:ar, men när knappt heller ICA-kassörskan ingår i mitt "umgänge" är det rätt konstigt. Men det är bara att hålla i. Jag har delvis viss insyn i vården och belastningen där har verkligen ökat i takt med den snabba smittspridningen som vi har nu i oktober/november. Min rädsla handlar nog annars fortsatt om att själv bli sjuk, att någon i familjen (som bor på annan ort då eftersom jag inte träffar dem) blir sjuk, någon vän eller att många andra blir det så vården inte mäktar med. Fasan vore att hamna i läget där det inte finns vård till alla som egentligen hade behövt. Hoppas vi slipper uppleva det i höst.		Kvinna	1970

					Sen är det så klart en del oro kring att människor förlorar jobb, mår mycket sämre än jag i mina grå hörn, får en kraschad ekonomi och att det ökar oron i samhället, fler brott, fler som behöver psykiatrisk vård, tar livet av sig, barn som far illa, folk som misshandlar sig ur sin frustration eller de som bara skiter i hela pandemin för att de inte orkar att hålla i och hålla ut.<br>Så det gäller att äta elefanten i små delar för att kunna få den i sig, ta en stund i taget, fokusera på det som funkar och vilka möjligheter som finns och inte fastna i allt det vi inte kan göra som vi gjorde innan. Allt vi gjorde innan kanske vi faktiskt inte ska hålla på med. Konstiga tider. Vi behöver varandra, men ska just nu inte vara nära varandra och ändå vara just nära, för det är nära som vi bryr oss om varandra.<br>Så tack för frågan och för att jag fick sätta mig och reflektera en stund. Bara hör av er igen om ni behöver fler tankar. Jag ställer upp!			

350	20200610	Jag är inte rädd för att bli sjuk, händer det så händer det. Men vad som kan göra mig rädd är att covid-19 verkar kunna slå över så fort och att då vara ensamstående och ligga hemma själv, den tanken kan göra mig rädd.	Jag går inte omkring och är rädd, det är ju inte lönt. Men jag förmodar att hade jag blivit sjuk hade nog rädslan kunnat gå över till en panikkänsla.	Inte jättemycket, jobbar med barn så "tydligen" är risken väldigt liten för mig att bli sjuk. Märkligt resonemang tycker jag, vi träffar ju kollegor, föräldrar och ibland måste jag åka stadsbussen till jobb. Det som har förändrats mest är mitt uteliv, jag väljer vilka ställen jag går till och helst ska det gå att sitta ute med glest mellan borden.	Jag är mest rädd för att den lättnad i restriktioner man kunde skönja inom en snar framtid åter kommer att stramas åt, pga av den ökning vi sett den senaste veckan. Jag vill ha tillbaka mitt spontan liv där jag bara kunde bestämma att ikväll går jag på bio eller till helgen tar jag bussen till landet. Även om vi inte har haft en fullskalig lock down, känns det som ens liv har fått sättas i isolering, den känslan har inte varit bra för mitt mående, för den har inneburit mycket ensam tid.		Kvinna	1963
351	20200610	Att bli smittad och mycket sjuk.	Jag känner en oro, för hälsan och framtiden. Oron gör att jag känner mig rastlös, då måste jag ta och göra något med mina händer, tex fixa och förändra hemma, skapa, renovera etc.	Följer råden och förhållnings reglerna som vi fått. Undviker större folksamlingar, affärer med mycket människor osv.		Sommaren kom som ett andningshål men min rädsla nu är den att mina nära och kära ska drabbas då sjukdomen kryper närmare och allt fler blir drabbade.<br>Själv jobbar jag i barnomsorgen och har svårt att hålla distans till alla jag träffar dagligen, det är också frustrerande och skrämmande.	Kvinna	1955

353	20200610	Jag är rädd för att bli smittad och drabbas hårt, på grund av min övervikt och av att jag var en rökare. Jag är rädd för att tvingas lämna mina barn med pappan för att vårdas på sjukhus/ eller dör. Jag är rädd för mina barn. Var ska de vara ifall jag och min man blir smittade tillsammans. Vi har inga släktingar.	Försöker hitta nyheter som visar hur många har lyckats med att besegra viruset. Ringa min mamma eller mina syskon(bor i USA) och berätta om mina känslor. Promenera för att se hur människor beter sig på ett ganska normalt sätt. Att de inte alla är livrädda som mig. Att inte alla är sjuka och ska dö i slutet.	- Har slutat träffas med vänner. Det är bara telefon och sociala medier som gäller. - Handlar i bestämma tider. Om det ser trångt ut i parkeringen utanför marknaden/köpcentrum, då återkommer jag vid en annan tid. - Tvättar (ibland använder jag handsprit) allt jag köper. - Låter inte barn leka i allmänna park, särskilt när det är mycket folk. - Aldrig äta i restaurangen(har läst om det första smittan i en restaurang och blivit rädd). - Aldrig använder kollektivtrafiken. Och i förebyggande syfte har jag: - slutat röka. Jag är rökfri i 3 månader nu. - ändrat mina matvanor och gått ner i vikt, för jag haft fetma med grad 2. - promenera varje dag.(har beställt en löpband för mer rörelser).	I början av epidemin var jag rädd för att bli smittad eftersom jag var en överviktig och rökig person. Så jag fattade ett avgörande beslut; Jag har slutat röka och genomgått en gastric sleeve operation. Men jag/ min man är fortfarande rädda för att drabbats av Corona, trots att vi följer alla säkerhetsrekommendationer. Vi är rädda att bli tvungna att hamna i sjukhuset och lämna våra döttrar själva (Jag är mamma till tre flickor. Den stora är tio år gammal). Vi har inga släktingar här i Sverige.		Kvinna	1981

364	20200729	Att bli smittad. Det är så få människor som håller avstånd, kanske 1 av 20.	I det här fallet arg och uppgiven.	Ja. Åker inte vissa bussturer. Går inte på restaurang. Går inte så mycket i affärer (förutom mataffärer). Har ändrat tiden jag åker buss hem från jobbet. Sätter mig ytterst på bussen så ingen kan sätta sig bredvid mig.	Jag är mest rädd för att smittas när jag vistas bland andra människor. Ytterst sällan upplever jag att andra håller avstånd, inte ens en halv armlängds. På jobbet ser jag nästan aldrig någon tvätta händerna när de kommer in i lokalerna trots att de åkt hiss och tagit i dörrhandtag.		Kvinna	1951
